# Genomic Expansions in the Human Gut Microbiome

**DOI:** 10.1093/gbe/evab156

**Published:** 2021-07-11

**Authors:** Andrew H Moeller

**Affiliations:** Department of Ecology and Evolutionary Biology, Cornell University, Ithaca, New York, USA

**Keywords:** adaptation, microbiota, population genetics, phylogenomics, phylogenetics

## Abstract

Bacteria inhabiting the human body vary in genome size by over an order of magnitude, but the processes that generate this diversity are poorly understood. Here, we show that evolutionary forces drive divergence in genome size between bacterial lineages in the gut and their closest relatives in other body sites. Analyses of thousands of reference bacterial isolate genomes and metagenome-assembled genomes from the human microbiome indicated that transitions into the gut from other body sites have promoted genomic expansions, whereas the opposite transitions have promoted genomic contractions. Bacterial genomes in the gut are on average ∼127 kb larger than their closest congeneric relatives from other body sites. Moreover, genome size and relative abundance are positively associated within the gut but negatively associated at other body sites. These results indicate that the gut microbiome promotes expansions of bacterial genomes relative to other body sites.

Significance

The bacteria inhabiting the human body vary in genome size by over 10-fold, but the causes of this variation are poorly understood. This study shows that bacteria inhabiting the gut microbiome have repeatedly evolved larger genomes compared with their closest relatives from other body sites. These results indicate that the human gut microbiome promotes genomic expansions in bacteria.

The human body is colonized by trillions of bacteria (Sender et al. 2016), some of which are descended from ancient symbionts that have been evolving within the human lineage for millions of years (Moeller, Caro-Quintero, et al. 2016). Recent work has shown that bacterial genome size differs systematically across human body sites, with a mean bacterial genome size in the gut nearly twice that in other body sites (Nayfach and Pollard 2015). This discrepancy could result from ancestral variation in genome size that was maintained after bacterial lineages transitioned into residency at their respective body sites. However, an untested nonmutually exclusive possibility is that evolutionary forces have generated divergence in bacterial genome size between the gut and other body sites in situ. Here, we leveraged genomic sequences generated from bacterial isolates (*n* = 2,206) (Human Microbiome Jumpstart Reference Strains Consortium 2010; Methé et al. 2012) and human metagenomes (*n* = 154,723 metagenome-assembled genomes [MAGs] representing 4,930 species-level genome bins) (Pasolli et al. 2019) to test whether the gut microbiome promotes expansions of bacterial genomes relative to other body sites.

First, we employed a comparative phylogenetic approach using genome sequences of bacteria isolated from different human body sites to test for associations between genome size and body site independent of bacterial evolutionary history. We generated a phylogeny of all high-quality (>90% complete) reference genomes from the HMP (supplementary data file 1, Supplementary Material online) for which curated body site metadata were available (supplementary table S1, Supplementary Material online). The HMP reference genome phylogeny contained 59 sets of congeneric bacterial lineages in which lineages isolated from the gut and lineages isolated from other body sites were reciprocally monophyletic. Each of these sets provides a phylogenetically independent test of whether evolutionary forces within the gut or other body sites have promoted divergence in genome size between bacterial lineages. A pruned phylogeny displaying relationships among the 59 comparisons on which downstream analyses were focused is presented in supplementary figure S1, Supplementary Material online. In 45 out of these 59 comparisons, bacterial lineages isolated from the gut displayed larger genomes than their closest relatives isolated from other body sites (sign test *P*-value = 3.3e-5). Gut-isolated genomes in these comparisons were on average 4.58% (127,405 bp) larger than the most closely related genomes derived from other body sites (fig. 1*A*, supplementary table S2, Supplementary Material online) (95% confidence interval: 1.74–7.41%). This differences in genome size became increasingly evident after correcting genomes for assembly completeness as estimated by CheckM (Parks et al. 2015): Genomes isolated from the gut were estimated to be 4.68% larger than conspecific genomes from other body sites (95% confidence interval: 1.99–7.37%). The difference in genome size between congeneric bacteria from the gut and other body sites was also evident when considering only the bacterial lineages detected at appreciable relative abundances in their respective body sites by shotgun metagenomic data (supplementary materials and methods, Supplementary Material online). Several predominant gut bacterial genera, such as *Enterococcus*, *Prevotella*, and *Lactobacillus*, contained multiple parallel divergences in genome size between strains isolated from the gut and strains isolated from other body sites (fig. 1*B*–*D*).

**Fig. 1.**
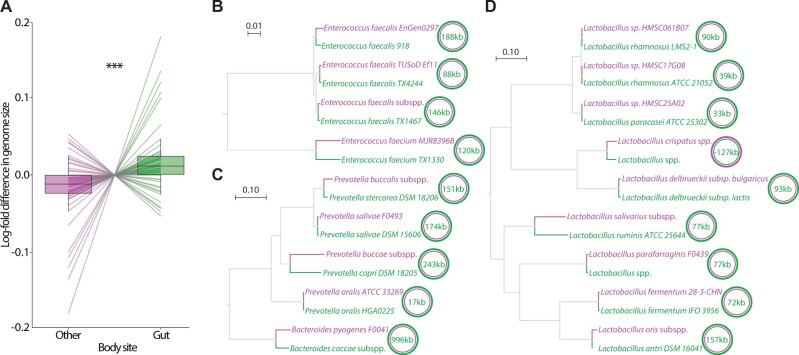
(*A*) Parallel divergence of bacterial genome sizes between the gut and other body sites. Box and whisker plots show the median log-fold difference in genome size between bacterial lineages isolated from the gut (green) and their closest congeneric relatives isolated from a nongut body site (purple). Each colored line represents one of 59 phylogenetically independent comparisons between congeneric lineages discordant for body site. Boxes delineate innerquartile ranges, and whiskers indicate maximum and minimum. Asterisks denote significance of difference in mean; ***P-value < 0.001. Phylogenies show all of the independent comparisons between congeneric lineages discordant for body site within the genera *Enterococcus* (*B*), *Prevotella* (*C*), and *Lactobacillus* (*D*). Scale bars indicate amino acid substitutions. Tip labels in green or purple denote isolates from the gut or a nongut body site, respectively. Tips representing multiple isolate genomes from the same subspecies or species are indicated by labels containing “subspp.” or “spp.,” respectively. Green and purple colored circles represent bacterial genomes from the gut or another body site, respectively. Circles are nested based on genome size, with the larger genome encircling the smaller genome. The difference in genome size between the strains from the gut and the other body site is shown in kilobases (kb) within each pair of circles. The body sites from which strains were isolated are presented in supplementary table S2, Supplementary Material online.

The divergence in genome size between congeneric bacteria from the gut and other body sites, with a trend toward larger genomes in the gut, was observed for each body site examined (i.e., airways, skin, oral cavity, and urogenital tract) (supplementary table S2, Supplementary Material online). Most of the phylogenetically independent comparisons of closely related genomes from different body sites included genomes from the oral cavity (*n* = 25) or genomes from the urogenital tract (*n* = 29). This observation is consistent with previous evidence of bacterial transmission into the gut from the oral cavity (Schmidt et al. 2019) and between the gut and urogenital tract (Freitas and Hill 2018). Genomes from both of these body sites were smaller on average than the most closely related genomes from the gut, but the oral cavity displayed the most significant trends. Of the 25 comparisons between oral and gut genomes, 22 displayed larger genomes in the gut (sign test *P*-value = 1e-4), with the CheckM-corrected mean genome size of gut genomes on average 8.83% larger (95% confidence interval: 3.25–14.4%). Of the 29 comparisons between urogenital and gut genomes, 19 displayed larger genomes in the gut (sign test *P*-value = 0.068), with the CheckM-corrected mean genome size of gut genomes on average 1.9% larger (95% confidence interval: −0.13% to 3.84%). Airways and skin were represented by only three comparisons each, but bacteria from these body sites also displayed smaller genomes on average than closely related lineages from the gut (supplementary table S2, Supplementary Material online). Overall, these results demonstrate a significant effect of transitions between the gut and other body sites (in particular, the oral cavity and urogenital tract) on bacterial genome size, leading to larger bacterial genomes in the gut compared with other body sites.

Many of the congeneric bacterial genomes from different body sites represent different bacterial species, which diverged in the more distant past than the timescales of human evolution. To assess the effects of body sites on bacterial evolution over shorter timescales, we next compared only the genomes of strains from the same bacterial species (i.e., conspecifics) isolated from different body sites. Comparisons included 24 cases in which closely related strains from the same bacterial species transitioned between the gut and another body site. The genomes of strains isolated from the gut were larger than the genomes of conspecifics isolated from other body sites in 17 out of the 24 comparisons (sign test *P*-value = 0.031), with a mean increase in genome size of 1.65% (supplementary table S2, Supplementary Material online) (95% confidence interval: 2e-4% to 3.43%). This differences in genome size became increasingly evident after correcting genomes for assembly completeness as estimated by CheckM (Parks et al. 2015): Genomes isolated from the gut were estimated to be 2.06% larger than conspecific genomes from other body sites (95% confidence interval: 0.666–3.47%). These results indicate repeated parallel divergence in genome size between bacterial lineages from the gut and conspecifics from other body sites.

The parallel divergence of bacterial genome size between the gut and other body sites could be due to genomic expansions in the gut or to genomic reductions at other body sites. To test for genomic expansions in the gut, we identified instances on the HMP isolate phylogeny in which gut-isolated lineages were nested within clades of lineages isolated from other body sites. The phylogeny contained 21 instances of this phylogenetic pattern, in which living in the gut appears to be the derived rather than the ancestral state. In 16 of the 21 comparisons, strains in the gut displayed larger genomes than their closest relatives from other body sites (sign-test *P*-value = 0.0133), with a mean increase in genome size in the gut of 2.87% (supplementary table S2, Supplementary Material online) (95% confidence interval: −0.59% to 6.34%). Circle diagrams displaying regions of similarity between genomes included in these comparisons are presented in supplementary figure S2, Supplementary Material online. We also conducted the reciprocal tests of whether transitions from the gut to other body sites have coincided with genome reduction. Bacterial genomes from nongut body sites were smaller than their closest relatives in the gut in 10 out of the 15 comparisons in which living at nongut body sites appeared to be the derived state (sign test *P*-value = 0.15), with a mean decrease in genome size of nongut lineages relative to gut lineages of −5.8% (95% confidence interval: 0.4% to −12%). These comparisons support a tendency for strains to evolve larger genomes after transitioning into the gut environment from another body site, and a weaker tendency for strains to evolve smaller genomes after transitioning out of the gut to another body site. Analyses based on genome sizes corrected by CheckM completeness estimates yielded similar results, as did phylogenetic ANOVA (Rohlfs and Nielsen 2015) (supplementary materials and methods, Supplementary Material online). Cumulatively, these results support the hypothesis that evolutionary forces operating in the gut have driven genomic expansions in multiple distantly related bacterial clades.

In addition to testing for genomic expansions in the gut microbiome relative to other body sites based on analysis of high-quality isolate genomes, we also tested whether this pattern was evident in analyses of MAGs. We filtered 154,723 MAGs generated from 47 metagenomic surveys of the human microbiome (Pasolli et al. 2019) for high-quality (>90% complete) genomes belonging to species-level genome bins detected in the gut and at least one other body site (supplementary materials and methods, Supplementary Material online). This filtering step identified 23 phylogenetically independent intraspecific comparisons capable of testing the hypothesis that the gut microbiome promotes genomic expansions relative to other body sites. In 16 out of 23 of these comparisons, genomes isolated from the gut were on average larger than genomes isolated from other body sites (sign test *P*-value = 0.0466), with a mean increase in size of 1.39% (95% confidence interval: −1.32% to 3.95%) (supplementary fig. S3, Supplementary Material online). The trend toward larger genomes in the gut than in other body sites was observed in each body site examined (supplementary table S3, Supplementary Material online). Results of these analyses lend further support to the conclusion that the gut microbiome promotes genomic expansions relative to other body sites.

Gut bacterial genomes were larger than their closest relatives from other body sites in the majority of phylogenetically independent comparisons, but in some comparisons gut bacterial genomes were smaller than their closest relatives at other body sites (supplementary table S2, Supplementary Material online). Five sets of bacterial lineages contain species under active pathogen surveillance by the Foodborne Diseases Active Surveillance Network (FoodNet) or the Foodborne Disease Outbreak Surveillance System (FDOSS) (Scallan et al. 2011). All five of these sets of lineages displayed smaller genomes in the gut than at other body sites, including *Campylobacter* sp., *Clostridium perfringens*, *Streptococcus* spp., *Escherichia coli*, and *Staphylococcus* sp. (supplementary fig. S4, Supplementary Material online). The probability of observing this pattern by chance is 0.075% given that 76.3% of comparisons displayed the opposite direction of genome-size divergence. Exclusion of pathogenic lineages under active surveillance by FoodNet or FDOSS increased support for the association between transitions into the gut from another body site and the evolution of larger genomes (supplementary materials and methods, Supplementary Material online). Gut bacterial lineages displayed larger genomes than closely related lineages from other body sites in 16 out of the 19 the remaining comparisons in which living in the gut appears to be the derived state (sign test *P*-value = 4.0e-4), with a mean increase in genome size of 4.10% (95% confidence interval: 0.81–7.40%) (fig. 2). These results suggest distinct evolutionary trajectories in the gut microbiome for the genomes of bacteria that display pathogenic tendencies and those of bacteria that display commensal or mutualistic tendencies.

**Fig. 2.**
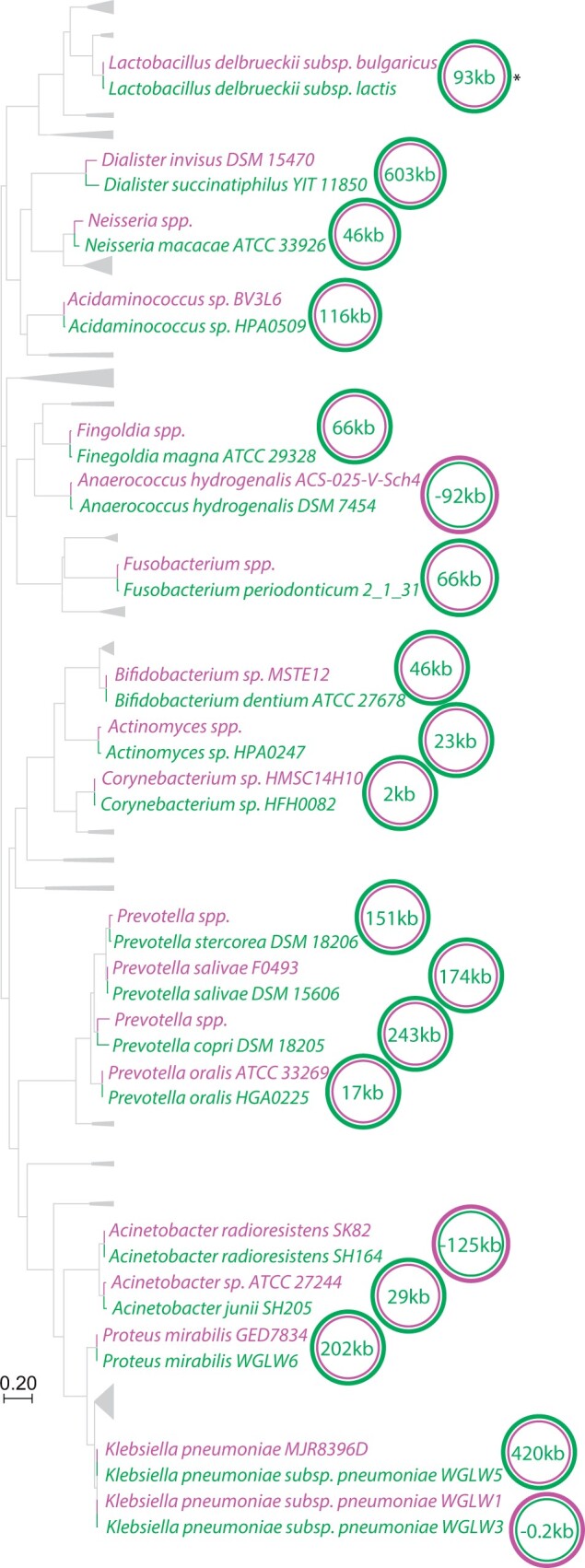
Parallel genomic expansions in the gut microbiome. Phylogeny shows the relationships among the comparisons of sibling congeneric bacterial lineages discordant for body site for which living in the gut microbiome was inferred to be the derived state, excluding pathogenic species tracked by FoodNet and FDOSS (supplementary fig. S3, Supplementary Material online). Collapsed clades correspond to other comparisons shown in supplementary figure S1 and table S2, Supplementary Material online. Tip labels in green or purple denote isolates from the gut or a nongut body site, respectively. Tips representing multiple isolate genomes from the same subspecies or species are indicated by labels containing “subspp.” or “spp.,” respectively. Green and purple colored circles represent bacterial genomes from the gut or another body site, respectively. Circles are nested based on genome size, with the larger genome encircling the smaller genome. The difference in genome size between the strains from the gut and the other body site is shown in kilobases (kb) within each pair of circles. Asterisk indicates comparison also shown in figure 2.

We next conducted gene and functional enrichment analyses to determine what types of DNA sequences underlie differences in genome size between bacterial lineages from the gut and their closest relatives from other body sites. These analyses allowed us to interrogate the parallel evolutionary divergence of gene functional content in phylogenetically matched pairs of genomes from the gut and another body site. We employed gene family and metabolic pathway analyses in Anvi’o (Eren et al. 2015) to identify the gene and functional content most enriched in genomes from the gut compared with their closely related genomes from other body sites. These analyses were based on Prodigal, COG, Pfam, TIGRFAM, KEGG Module, and KEGG Class annotations (supplementary tables S4–S8, Supplementary Material online). Results indicated that most of the functions with nonzero enrichment scores were more abundant in the gut than in other body sites. For example, 254 KEGG Modules displayed positive enrichment scores for the gut compared with only 118 KEGG Modules with positive enrichment scores for other body sites (sign test *P-*value = 5.72e-5). Similar results were also observed when comparing only closely related pairs of genomes from the gut and oral body sites (supplementary tables S9–S13, Supplementary Material online). Analyses also identified functional divergence between closely related genomes that may be ecologically relevant for bacterial lifestyles in gut and nongut body sites. For example, the genome of *Lactobacillus ruminis* (NCBI accession number: ASM15937v2) from the gut contains genes coding for sugar, oligopeptide, phosphate, and iron transporters (TIGRFAM annotations, supplementary table S14, Supplementary Material online) not found in the genome of closely related *Lactobacillus* lineages (e.g., NCBI accession number: ASM17947v1) from other body sites.

In addition, the genomes of bacteria from the gut contained mobile genetic elements and phage-associated regions not found in genomes from other body sites (supplementary fig. S2, Supplementary Material online), although these regions did not account for all of the regions in genomes from the gut not found in closely related genomes from other body sites. In particular, genes associated with CRISPR systems were overrepresented among annotations displaying nonzero enrichment scores between genomes from the gut and other body sites. For example, only 3 out of the 3,674 Prodigal-annotated gene families displaying enrichment scores of zero were associated with CRISPR systems. In contrast, 9 out of the 2,210 gene families displaying positive enrichment scores in gut-isolated genomes (Fisher’s exact test, *P*-value = 0.013) and 19 out of 1,829 gene families displaying positive enrichment scores in genomes derived from other body sites were CRISPR-associated genes (Fisher’s exact test, *P*-value < 0.0001). The divergence of CRISPR regions between genomes from the gut and congenerics from other body sites is consistent with previous observations that these regions can vary substantially even among closely related bacterial genomes (Deveau et al. 2010). These results are also consistent with a history of parallel acquisition of CRISPR regions among distantly related bacterial lineages within human body sites. However, no individual gene family or functional annotation was significantly enriched in either genomes from the gut or genomes from other body sites after correction for multiple testing (supplementary tables S4–S13, Supplementary Material online), even when tests were performed on subsets of related annotations (supplementary materials and methods, Supplementary Material online). Together, these results suggest that the repeated expansions of bacterial genomes in the gut microbiome have been underlain by a diversity of distinct sets of gene families in different bacterial lineages.

In addition to functional content, we also tested whether other genomic features, such as GC content, coding density, and codon usage frequencies, differed significantly between phylogenetically matched pairs of genomes from the gut and other body site. Results indicated no consistent differences in these genomic features as a function of body site. Details of these analyses are presented in the supplementary materials and methods, Supplementary Material online.

Genomic expansions in the gut microbiome could be driven by multiple distinct, nonmutually exclusive evolutionary forces. To assess whether natural selection may be contributing to the genomic expansions of gut bacterial lineages, we tested for an association between genome size and bacterial fitness as measured by relative abundance estimates from HMP metagenomic data sets (Segata et al. 2012; Nayfach and Pollard 2015). These analyses revealed a positive association between the mean genome size and mean relative abundance of bacterial species within individual human gut microbiomes (*R*^2^ = 0.21; *P*-value = 9.2e-10), but negative associations at other body sites (fig. 3) (supplementary table S15, Supplementary Material online). In the gut microbiome, genome size alone explained 21% of the variation in the mean relative abundances of bacterial species within individual hosts. The positive relationship between genome size and relative abundance of bacterial species was also observed within individual bacterial genera (*R*^2^ = 0.024; *P*-value = 0.042) and by regression of phylogenetically independent contrasts (Felsenstein 1985) (*R*^2^ = 0.06; *P*-value = 0.0011) (supplementary fig. S5, Supplementary Material online), indicating that the association between genome size and relative abundance is evident within disparate clades of the bacterial phylogeny. The observations suggest selective forces generating and maintaining larger genome sizes in the gut microbiome relative to other body sites.

**Fig. 3.**
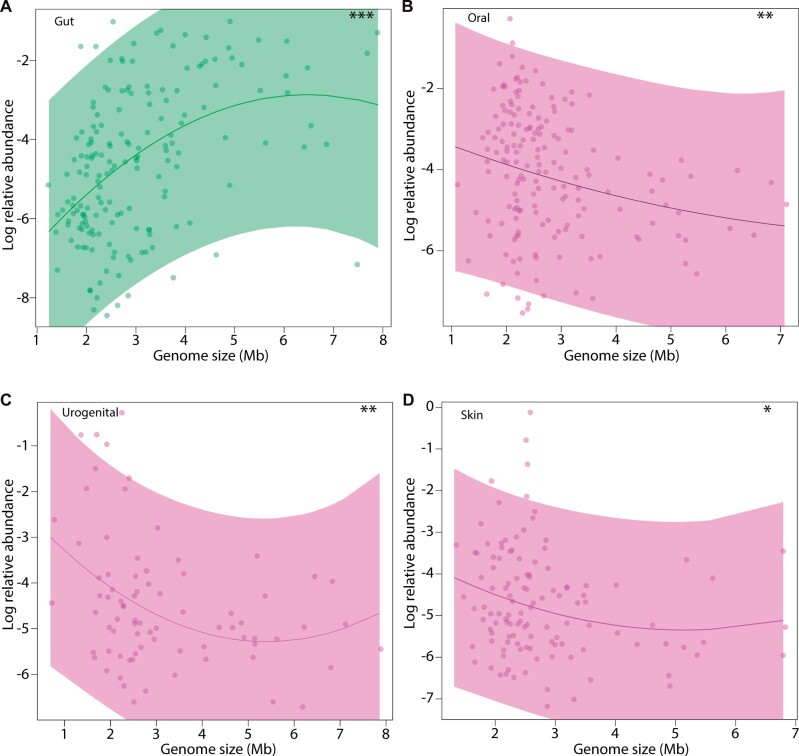
Associations between genome size and relative abundance within human body sites. Curves show best-fit polynomial regressions between average genome sizes of species and their relative abundances in body sites of the human microbiome. Each point represents a bacterial species detected in the gut (*A*), oral cavity (*B*), urogenital tract (*C*), or skin (*D*) by metagenomic shotgun sequencing. Shading represents 95% confidence interval for regression curves. The *x*-axis shows the average genome size of each species in megabases (Mb). The *y*-axis shows the mean log-relative abundance of species within individual humans. Asterisks show significance of linear coefficient from polynomial regression analysis; *P-value < 0.05, **P-value < 0.01, ***P-value < 0.001.

Other studies of genome evolution in symbiotic bacteria of eukaryotes have observed trends toward genomic reduction, rather than genomic expansion. For example, obligate endosymbionts of animals typically display reduced genomes, the smallest known of which contains only ∼112 kb (Bennett and Moran 2013) (not counting organelle genomes), and the obligate intracellular pathogens of mammals tend to evolve smaller genomes (Moran 2002). These genomic reductions can be attributed at least in part to genetic drift (Mira et al. 2001): Most bacteria display mutational biases toward deleting DNA, and obligate endosymbionts undergo bottlenecks during transmission between hosts that reduce effective population sizes (*N*_e_). Together, these forces lead to reduced efficacy of selection, increased influences of mutational biases and the erosion of genomes (McCutcheon and Moran 2011). In contrast, bacteria within the human gut microbiome are less likely to experience strong genetic drift. Gut bacterial population sizes can reach trillions of cells (Sender et al. 2016), and lineages are readily transmitted among hosts within individual host generations (Tung et al. 2015; Moeller, Foerster, et al. 2016; Brito et al. 2019). Moreover, the highly competitive and energy-rich gut environment may favor bacterial lineages with large and functionally diverse gene repertoires. Under these demographic and ecological scenarios, the evolutionary forces appear to have promoted the expansion of bacterial genomes.

## Supplementary Material

Supplementary data are available at *Genome Biology and Evolution* online.

## Supplementary Material

evab156_Supplementary_Data
