# Supplementary material for: Genomic Expansions in the Human Gut Microbiome
Source: Genome Biol Evol. 2021 Jul 11;13(7):evab156. doi: 10.1093/gbe/evab156 (PMC8325571; doi:10.1093/gbe/evab156)
Supplement: evab156_Supplementary_Data [file evab156_supplementary_data.zip › SupplementaryMaterials_GenomeSize.docx]

**Supplementary Materials and Methods**

*Phylogenomic analyses*

We constructed a phylogenetic tree of all 2,206 genome assemblies from the Human Microbiome Project under NCBI bioproject accession PRJNA28331. Anvi'o (Eren et al., 2015) was used to concatenate aligned proteins from each genome in the Bacteria_71 collection. RAxML (Stamatakis, 2014) was used to infer bootstrapped maximum likelihood phylogenies from the alignment under the PROTGAMMAWAG model of substitution and 50 replicates. All comparisons of genome size were based on relationships supported by >80% of bootstrap replicates.

*Comparisons of genome size between bacterial lineages*

Body-site metadata for HMP reference genomes were obtained from the HMP catalog at https://www.hmpdacc.org/hmp/catalog/. These metadata and the maximum likelihood strain genome phylogeny were used to identify all pairs of sibling congeneric lineages (i.e., individual isolate genome or monophyletic clade of isolate genomes) for which one lineage was isolated from the gut and the other lineage was isolated from another body site. Genome sizes of isolates were obtained from NCBI and used to calculate the mean difference in genome size between lineages isolated from different body sites for each pair of lineages. For lineages represented by a monophyletic clade of multiple genomes derived from the same body site, the branch length weighted average genome size was used to calculate the mean difference in genome size between body sites. CheckM (Parks et al., 2015) was employed to estimate assembly completeness for each genome. All comparisons of genome size differences between body sites were repeated on completeness-corrected genomes to control for differences in completeness among assemblies (Table S2). In addition, we conducted a separate analysis focused only on comparisons containing bacterial lineages detected in their respective human body sites by shotgun metagenomic data at >1e-7 relative abundance (Abubucker et al., 2012).

*Comparisons of gene density, GC content, and codon usage*

Gene density and GC content was calculated for each pair of genomes in CheckM. Codon usage for each genome was calculated in Anvi’o using anvi-get-codon-frequencies. Differences between genomes from the gut and genomes from other body sites in these parameters were calculated for every pair of closely related genomes. Confidence intervals for the differences in each parameter between closely related genomes were constructed, with intervals not overlapping zero indicating significant differences between the gut and other body sites. *P*-values for comparisons of codon frequencies were adjusted for multiple testing by FDR correction.

*Inference of derived and ancestral bacterial habitats*

To test for associations between transitions into or out of the gut microbiome and changes in genome size, we identified comparisons of lineages in which living in the gut appeared to be either the derived or ancestral state. Gut dwelling was scored as the ancestral state for comparisons when the outgroup to the lineages contained within the comparison was isolated from the gut. Gut dwelling was scored as the derived state for comparisons when the outgroup to the lineages contained within the comparison was isolated from the non-gut body site. The ancestral state was not scored for comparisons whose outgroup lineage was derived from a different body site than those included in the comparison, or for comparisons whose outgroup lineage contained a clade of genomes derived from more than one body site.

*Analyses of MAGs*

We filtered all Metagenome Assembled Genomes (MAGs) from Pasolli et al., (2019) to identify high-quality (>90% complete) genomes from Species-Level Genome Bins (SGBs) detected in the gut and either the oral or skin microbiomes (i.e., the non-gut body sites included in Pasolli et al, 2019). Genome sizes were corrected by completeness, and the average genome size within each body site was calculated for each SGB. Averages were then used to test the hypothesis that the gut microbiome promotes genomic expansion relative to other body sites. Theses analyses included 23 phylogenetically independent comparisons, the results of which are presented in Table S3.

*Differential abundance of bacterial genomic functional content between the gut and other body sites*

To identify the genomic functional content underlying differences in genome size between the gut and other body sites, Anvi’o was employed to test for gene and pathway enrichment (Clusters of Orthologous Groups - COGs, Prodigal, Pfam, TIGRFAM, KEGG Class, and KEGG Module) using the pan-genomics workflow and the function anvi-get-enriched-functions-per-pan-group. These analyses focused on matched pairs of closely related genomes from different body sites, such that each sample group (i.e., genomes from the gut or genomes from a non-gut body site) contained exactly 59 genomes, one from each phylogenetically independent comparison. For comparisons containing multiple bacterial genomes from a single body site, a random genome was selected. These analyses were conducted in ten replicates, with qualitatively similar results, and statistics from a randomly selected replicate are reported. To test whether differences in genome size were underlain by plasmids, enrichment analyses were reperformed on Prodigal annotations containg the word ‘plasmid’. In addition, Plasmid Finder (Carattoli et al., 2014) was employed to identify and remove contigs containing putative plasmid sequences in each pair of genomes and genome size comparisons were reperformed, yielding qualitatively similar results (i.e., no change in the direction of genome divergence in any comparison).

We also visualized the locations of CRISPR/Cas, phage, and mobile genetic elements in the gut bacterial genomes included in comparisons in which living in the gut was inferred to be the derived state. CRISPR/Cas elements were identified from NCBI RefSeq annotations, phage elements were identified using PHASTER (Arndt et al., 2016) under default settings, and mobile genetic elements were identified using MobileElementFinder (Johansson et al., 2021) under default settings. CRISPR/Cas, phage, and mobile genetic element were imported into BRIG (Alikhan et al., 2011) for ring visualization.

*Associations between genome size and relative abundance*

Relative abundances of named bacterial species detected in metagenomic shotgun sequencing of different body sites were obtained from MG-RAST (Meyer et al., 2008). In Figure 4, oral, skin, urogenital tract, and gut correspond to HMP body sites buccal mucosa, retroauricular crease, posterior fornix, and gastrointestinal tract, respectively. Average genome size for each species was calculated from HMP reference genomes when available and from NCBI accessions in cases where HMP reference genomes were not available. Linear and polynomial regression analyses were conducted in R to test for associations between the relative abundance of species and genome size within each body site. Likelihood ratio tests were employed to test whether polynomial regression significantly better explained the relationship between genome size and relative abundance for each body site than linear regression. Phylogenetically independent contrasts for the relative abundances and genome sizes of bacterial species were calculated using the package ‘ape’ (Paradis et al., 2004) in R.

**Supplementary Results**

*Genome size divergence between body sites*

In addition to testing for genome-size divergence across all pairs of genomes from congeneric bacteria discordant for body site, we also tested whether this pattern was evident for only pairs of genomes for which body site residency was supported by shotgun metagenomic data. First, we tested whether the divergence in genome size between the gut and non-gut body sites was evident in the subset of comparisons containing bacterial lineages belonging to species detected in at least one of their respective human body sites by shotgun metagenomic data at >1e-7 mean relative abundance across HMP study participants (736 samples) (Segata et al., 2012) as estimated by MetaPhlAn (Nayfach and Pollard, 2015; Segata et al., 2012). This analysis included a total of 28 comparisons, and relative abundance estimates for the species included in each comparison are presented in Table S2. In 20 out of these 28 comparisons, genomes from the gut were larger than the closely related genomes from other body sites (sign test *p*-value = 0.0178). In this subset of comparisons, genomes from the gut were on average 2.89% larger than the most closely related genomes from other body sites (95% Confidence Interval: 0.03% to 5.43%). This analysis included 10 comparisons for which both lineages included belonged to species detected at >1e-7 relative abundance at each of their respective body sites by shotgun metagenomic data (i.e., both members of the comparison were present in metagenomic data at appreciable abundances from their respective body sites). In 9 out of 10 of these comparisons, genomes from the gut were larger than the closely related genomes from other body sites (sign test *p*-value = 0.0107). In this subset of comparisons, genomes from the gut were on average 3.49% larger than the most closely related genomes from other body sites (95% Confidence Interval: 2.38% to 4.61%). These results indicate that the divergence in genome size between the gut and other body sites was evident even when only considering pairs of bacterial lineages for which body site residency was inferred by metagenomic data (as opposed to using the known body site source of bacterial isolates). The observation that the statistical significance of the difference in genome size between gut and non-gut body sites increased when tests were applied only to comparisons for which both species represented were detected at appreciable (>1e-7) relative abundances in metagenomic data (i.e., the 10 comparisons described above) further strengthens support for parallel changes in bacterial genome size within gut and non-gut body sites.

In addition to assessing the residency of isolated bacterial lineages at specific body sites with relative abundance estimates from MetaPhlAn, we also identified the subset of genomes in our comparisons for which metagenome assembled genomes (MAGs) have been recovered from human microbiome datasets (Pasolli et al., 2019). We used FastANI (Jain *et al.*, 2018) to calculate all pairwise average nucleotide identities (ANIs) between assemblies included in the comparisons (Table S2) and the representative set of MAGs from Pasolli et al. (2019), then filtered all comparisons for which both sets of genome assemblies displayed <98% ANI to every MAG. This filtering step yielded a set of 35 comparisons for which at least one bacterial lineage included in the comparison was detected as a MAG in a metagenome from the body site from which the lineage was isolated. These comparisons are indicated in Table S2. In 29 out of 35 of these comparisons, genome size was larger in bacterial isolated from the gut than in bacteria isolated from the other body site (sign test *p*-value = 4.72e-5). Genomes from the gut were on average 3.62% larger than genomes from other body sites (95% Confidence Interval: 1.61% to 8.85%). These comparisons included two instances in which representatives of both bacterial lineages were detected as MAGs in metagenomes from their respective body sites, and in both cases genomes from the gut were larger than genomes from the other body site. Note that only eight out of 46 of the metagenome studies analyzed by Pasolli et al., (2019) included samples from the gut; therefore, the lack of MAGs for many isolates from non-gut body sites may represent a lack of sampling rather than absence from the body site. Cumulatively, these results lend further support to the conclusion that the gut microbiome promotes genomic expansions in bacteria relative to other body sites.

*Genomic expansions in the gut and contractions at other body sites*

Analyses based on genome sizes corrected by CheckM completeness estimates further supported the conclusion the transitions into the gut from other body sites promotes genomic expansion. In 16 of the 21 comparisons in which living in the gut appears to be the derived rather than the ancestral state, completeness corrected genome size estimates of strains in the gut were larger than those of their closest relatives from other body sites (sign-test p-value = 0.0133), with a mean increase in genome size in the gut of 3.25% (Table S2) (95% Confidence Interval -0.24% to 6.27%). In 10 out of the 15 comparisons in which living at non-gut body sites appeared to be the derived state, completeness corrected genome size estimates of strains from the non-gut body site displayed a nonsignificant tendency to be smaller than those of their closest relatives from the gut (sign test p-value = 0.15), with a mean decrease in genome size of non-gut lineages relative to gut lineages of -7.6% (95% Confidence Interval 0.8% to -16%).

*Associations between body site and genome size independent of bacterial phylogenetic history*

Phylogenetic ANOVA (Rohlfs and Nielsen, 2015) was applied to test for an association between bacterial transitions between body sites and changes in genome size along all branches of the HMP isolate phylogeny (Supplementary Data File 1). These analyses were conducted in R in the geiger package (Pennell et al., 2014). Results indicated an association between living in the gut or other body sites and genome size independent of bacterial phylogenetic history (*p*-value = 0.0014)

*GC content, codon usage, and gene density*

In addition to genome size, we also tested for significant differences in GC content, codon usage, and gene density between closely related genomes from the gut and other body sites. The mean GC content was on average 0.78% higher and the coding density was on average 0.77% lower in genomes from the gut than in genomes from other body sites, and some variation in codon frequencies was observed. However, these analyses revealed no significant differences between genomes from the gut and genomes from other body sites (95% confidence intervals of fold change in frequency overlapping zero in every comparison).

*Differential abundance of bacterial genomic functional content between the gut and other body sites*

For comparisons in which living in the gut was inferred to be the derived state, several of the regions of differentiation between gut bacterial genomes and genomes from other body sites corresponded to CRISPR/Cas, phage, and mobile genetic elements (Figure S2), although not all regions of differentiation corresponded to these elements. Interestingly, the largest regions of differentiation (>100kb) did not contain CRISPR/Cas, phage, and mobile genetic elements, but were often located nearby these elements (Figure S2). These results suggest that genomic expansions in the gut have been mediated in part by acquisition of phage and other mobile genetic elements not found in closely related genomes from other body sites.

*Relationship between genome size and relative abundance*

Linear and polynomial regression analyses indicated that relative abundance and genome size were positively associated in the gut, but negatively associated at other body sites. Body-site specific relative abundances of bacterial species were estimated in MetaPhlAn from shotgun metagenomic data generated from 736 samples from the Human Microbiome Project. Genome size estimates for bacterial species were calculated as the average genome size of high-quality genomes for each species. Likelihood ratio tests of nested models were employed to assess whether polynomial or linear regression better explained the data for each body site. Likelihood ratio tests indicated that polynomial regression better explained the relationship between genome size and relative abundance for each body site.

**Supplementary References**

Alikhan NF, Petty NK, Zakour NL, Beatson SA. 2011. BLAST Ring Image Generator (BRIG): simple prokaryote genome comparisons. *BMC Genomics*. 12:1–0.

Arndt D, Grant JR, Marcu A, Sajed T, Pon A, Liang Y, Wishart DS. 2016. PHASTER: a better, faster version of the PHAST phage search tool. *Nucleic Acids Research*. 44:16–21.

Carattoli A, Zankari E, García-Fernández A, Larsen MV, Lund O, Villa L, Aarestrup FM, Hasman H. 2014. In silico detection and typing of plasmids using PlasmidFinder and plasmid multilocus sequence typing. *Antimicrobial Agents and Chemotherapy*. 58:3895–903.

Jain C, Rodriguez-R LM, Phillippy AM, Konstantinidis KT, Aluru S. 2018. High throughput ANI analysis of 90K prokaryotic genomes reveals clear species boundaries. *Nature Communications*. 9:1–8.

Johansson MH, Bortolaia V, Tansirichaiya S, Aarestrup FM, Roberts AP, Petersen TN. 2021. Detection of mobile genetic elements associated with antibiotic resistance in Salmonella enterica using a newly developed web tool: MobileElementFinder. *Journal of Antimicrobial Chemotherapy*. 76:101–109.

Meyer F, Paarmann D, D'Souza M, Olson R, Glass EM, Kubal M, Paczian T, Rodriguez A, Stevens R, Wilke A, Wilkening J. 2008. The metagenomics RAST server–a public resource for the automatic phylogenetic and functional analysis of metagenomes. *BMC Bioinformatics* 9:1–8.

Paradis E, Claude J, Strimmer K. 2004. APE: analyses of phylogenetics and evolution in R language. *Bioinformatics* 20:289–90.

Stamatakis A. 2014. RAxML version 8: a tool for phylogenetic analysis and post-analysis of large phylogenies. *Bioinformatics* 30:1312–3.

**Figure S1. Phylogeny of all congeneric sibling bacterial isolates discordant for body site from the Human Microbiome Project reference genomes.** Pruned phylogeny derived from Supplementary Data File 1 shows the relationship among sibling bacterial lineages belonging to the same genus but isolated from different body sites. All sibling relationships were supported by >50% of bootstrap replicates. Branch lengths indicate substitutions per site. Tip labels in green or purple correspond to isolates from the gut or a non-gut body site, respectively. Tips correspond to comparisons listed in Table S2.

**Figure S2. Visualization of genomic differences between isolates from the gut and non-gut body sites.** Ring diagrams show the regions displaying >70% sequence similarity between genomes derived from the gut (inner ring) and from another body site (out ring). Details for each comparison are presented in Table S2. Diagrams were generated with BRIG using default settings (Alikhan et al., 2011).

**Figure S3. Parallel divergence of bacterial genome sizes between the gut and other body sites based on analyses of MAGs.** Box and whisker plots show the median log-fold difference in genome size between Metagenome Assembled Genomes (MAGs) from the gut (green) and their closest relatives from the same Species-Level Genome Bin (SGB) assembled from a non-gut body site (purple). Each colored line represents one of 23 phylogenetically independent comparisons between conspecific lineages discordant for body site. Boxes delineate inner-quartile ranges, and whiskers indicate maximum and minimum. Asterisks denote significance of sign test; * *p*-value < 0.05.

**Figure S4. Lineages related to foodborne pathogens display smaller genome sizes in the gut than at other body sites.** Phylogeny shows the relationships among the comparisons of sibling congeneric bacterial lineages discordant for body site containing pathogenic species tracked by FoodNet and FDOSS. Tip labels in green or purple correspond to isolates from the gut or a non-gut body site, respectively. Tips representing multiple isolate genomes from the same subspecies are indicated by labels containing ‘subspp.’. Green and purple colored circles represent bacterial genomes from the gut or another body site, respectively. Circles are nested based on genome size, with the larger genome encircling the smaller genome. The difference in genome size between the strains from the gut and the other body site is shown in kilobases (kb) within each pair of circles.

**Figure S5. Positive association between bacterial genome size and relative abundance in the gut microbiome independent of bacterial evolutionary history.** A) Curve represents the best-fit polynomial regression between bacterial genome size and genus-normalized mean relative abundance in the human gut microbiome. Genus-normalized mean relative abundances for each species were calculated by dividing the mean relative abundance of each bacterial species by the mean relative abundance of that species’ congenerics. B) Green lines represent best-fit linear regression between phylogenetically independent contrasts (PICs) calculated for mean log relative abundance of bacterial species and mean genome size of bacterial species. PICs were calculated using a phylogeny containing bacterial species detected in the gut microbiome by metagenomic shotgun sequence and analyses in MG-RAST.

**Table S1. Metadata for HMP high-quality reference genomes**

**Table S2. Phylogenetically independent tests of genome size divergence between gut and other body sites based on isolate genomes.**

**Table S3. Phylogenetically independent tests of genome size divergence between gut and other body sites based on MAGs.**

**Table S4. Statistics for Prodigal and Pfam annotation enrichment between the gut and other body sites for phylogenetically matched bacterial lineages.**

**Table S5. Statistics for TIGRFAM annotation enrichment between the gut and other body sites for phylogenetically matched bacterial lineages.**

**Table S6. Statistics for KEGG module annotation enrichment between the gut and other body sites for phylogenetically matched bacterial lineages.**

**Table S7. Statistics for KEGG class annotation enrichment between the gut and other body sites for phylogenetically matched bacterial lineages.**

**Table S8. Statistics for COG annotation enrichment between the gut and other body sites for phylogenetically matched bacterial lineages.**

**Table S9. Statistics for KEGG class annotation enrichment between the gut and oral body sites for phylogenetically matched bacterial lineages.**

**Table S10. Statistics for KEGG module annotation enrichment between the gut and oral body sites for phylogenetically matched bacterial lineages.**

**Table S11. Statistics for Pfam annotation enrichment between the gut and oral body sites for phylogenetically matched bacterial lineages.**

**Table S12. Statistics for COG annotation enrichment between the gut and oral body sites for phylogenetically matched bacterial lineages.**

**Table S13. Statistics for TIGRFAM annotation enrichment between the gut and oral body sites for phylogenetically matched bacterial lineages.**

**Table S14. Annotations for pairs of congeneric genomes from different body sites.**

**Table S15. Statistics from regression analyses of genome size and relative abundance in skin, oral, urogenital, and gut microbiomes.**

**Supplementary Data File 1. Genome-resolved phylogeny of 2,206 bacterial isolates from the Human Microbiome Project.** Maximum likelihood phylogeny of all 2,206 bacterial reference genomes from the Human Microbiome Project was inferred in RAxML from an alignment of single-copy proteins from the Anvi’o Bacteria_71 collection.
