# Supplementary figures and images for: Genomic Expansions in the Human Gut Microbiome
Source: Genome Biol Evol. 2021 Jul 11;13(7):evab156. doi: 10.1093/gbe/evab156 (PMC8325571; doi:10.1093/gbe/evab156)

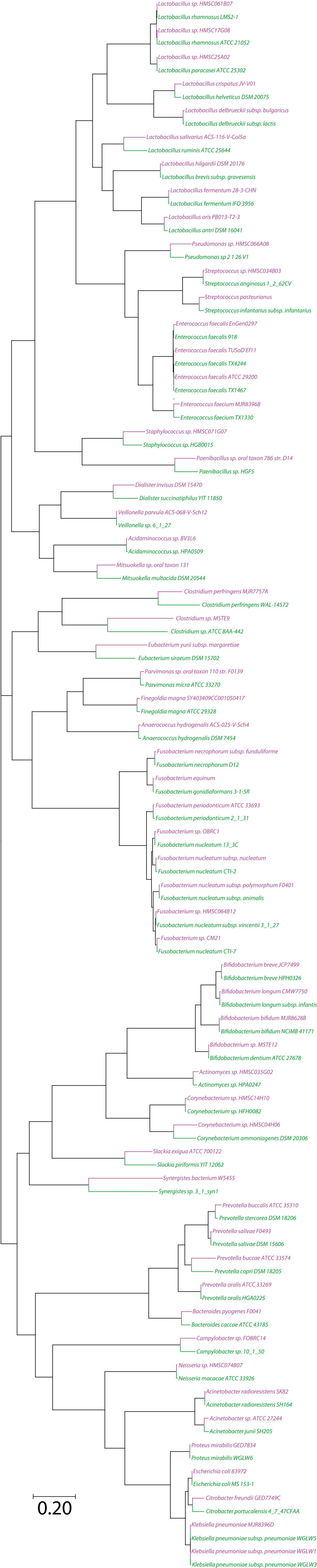

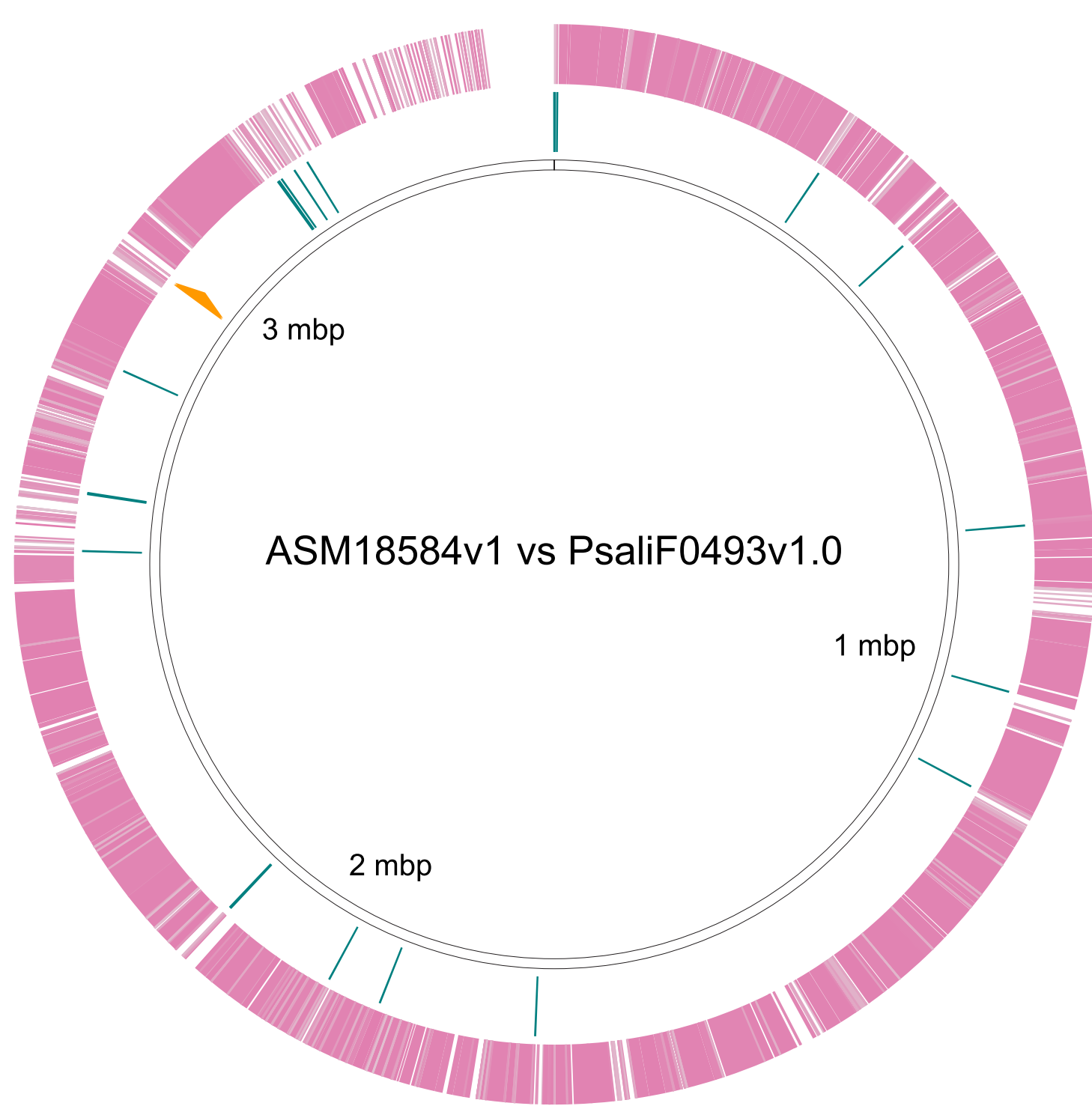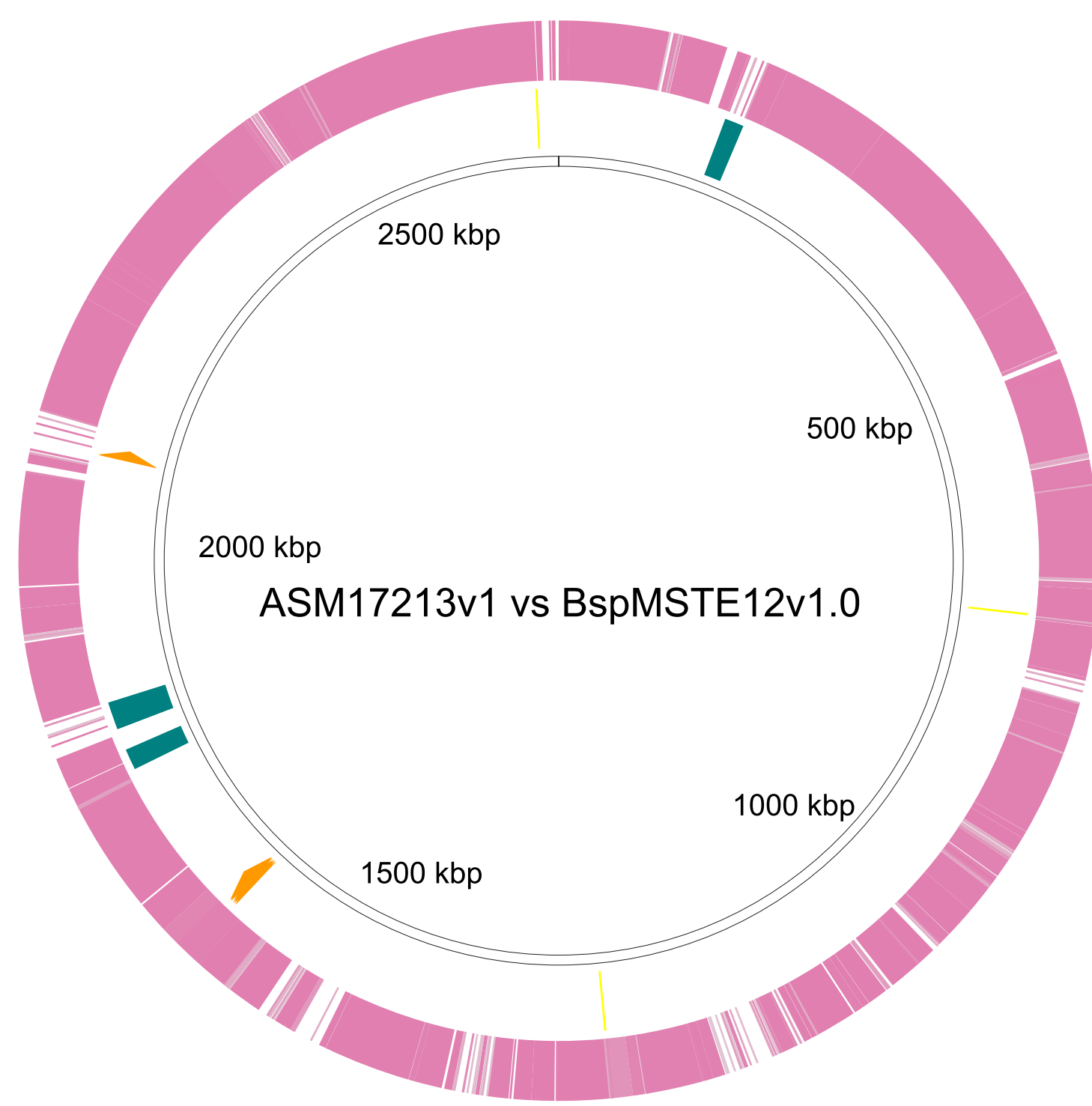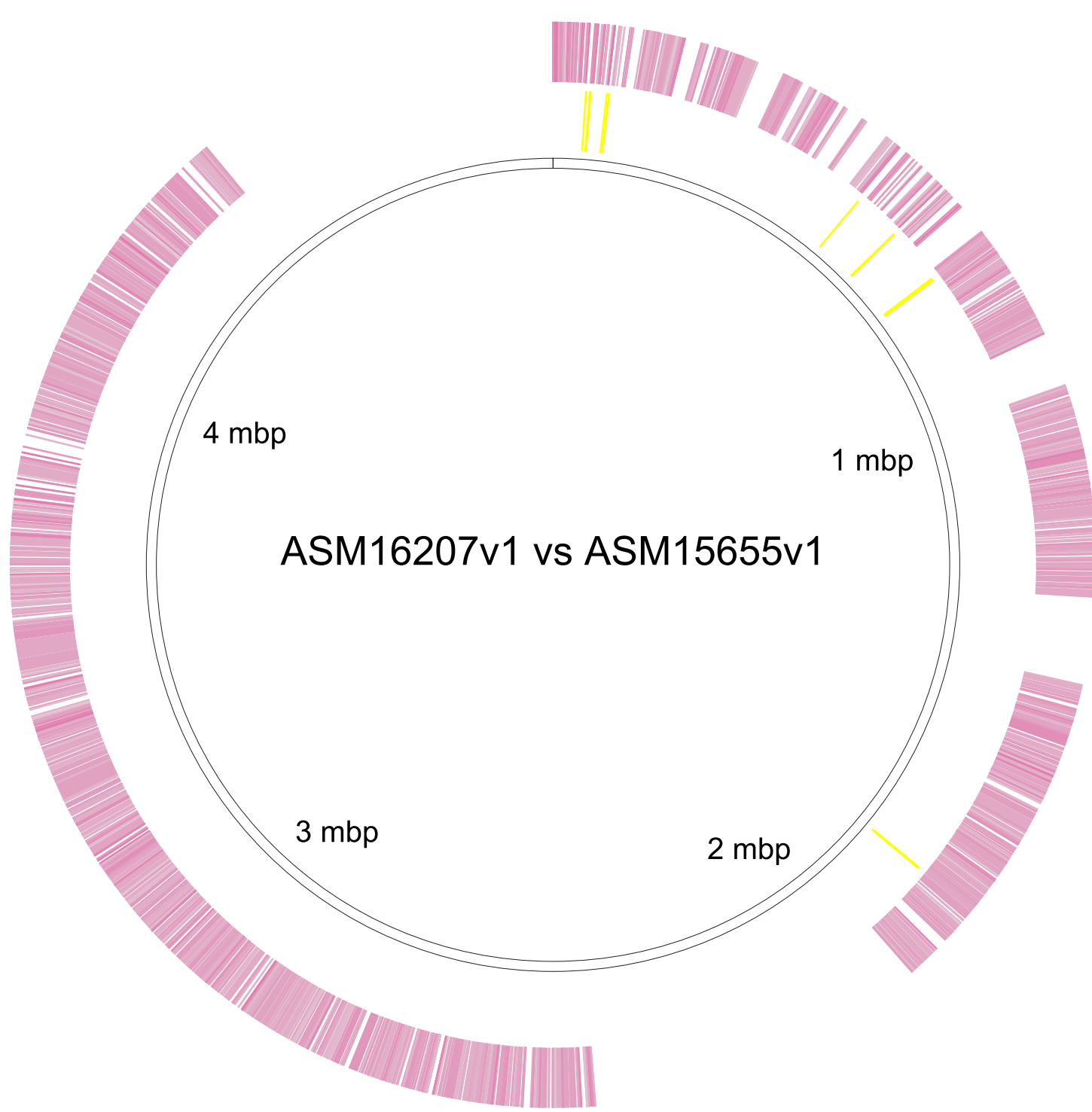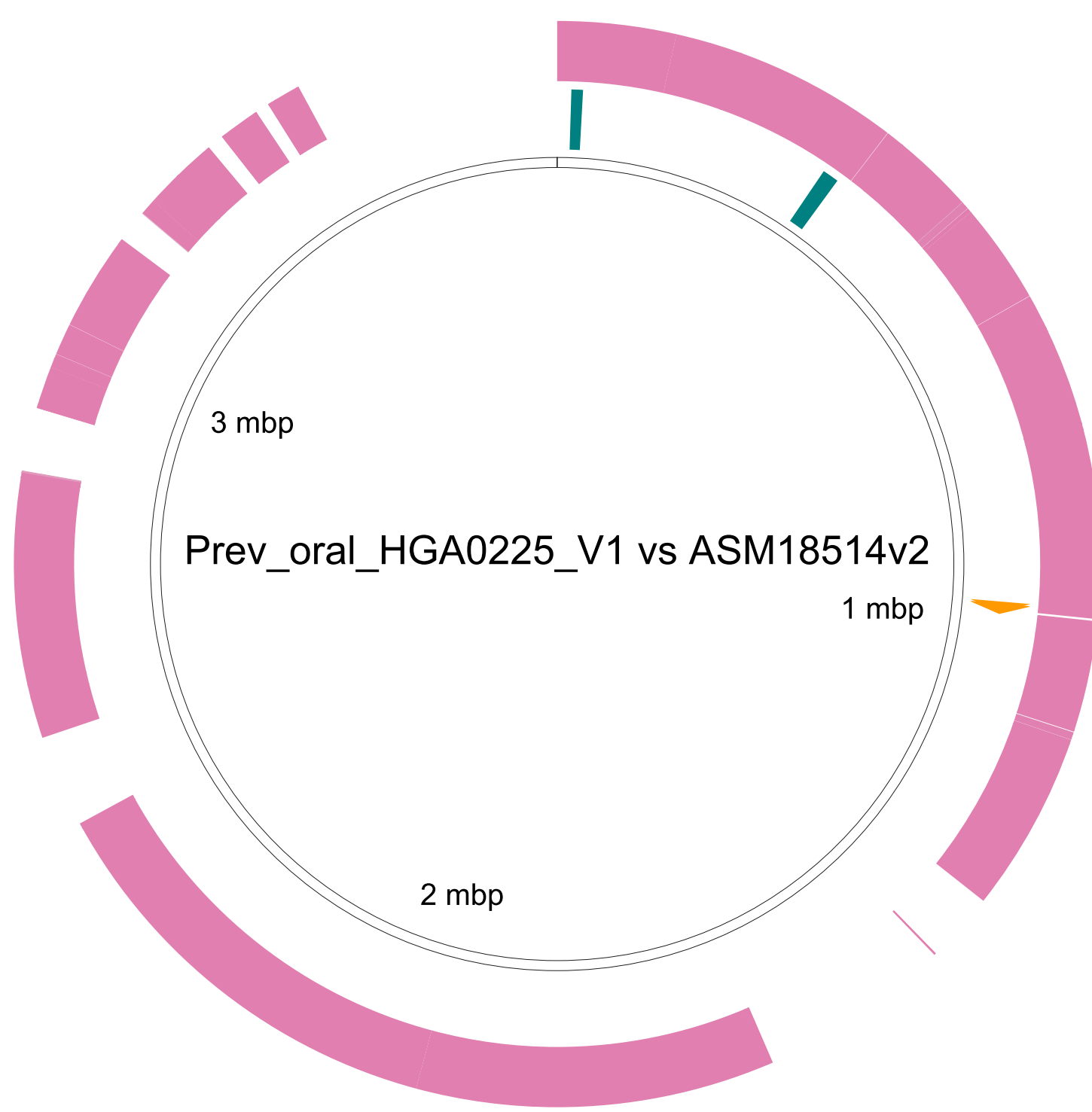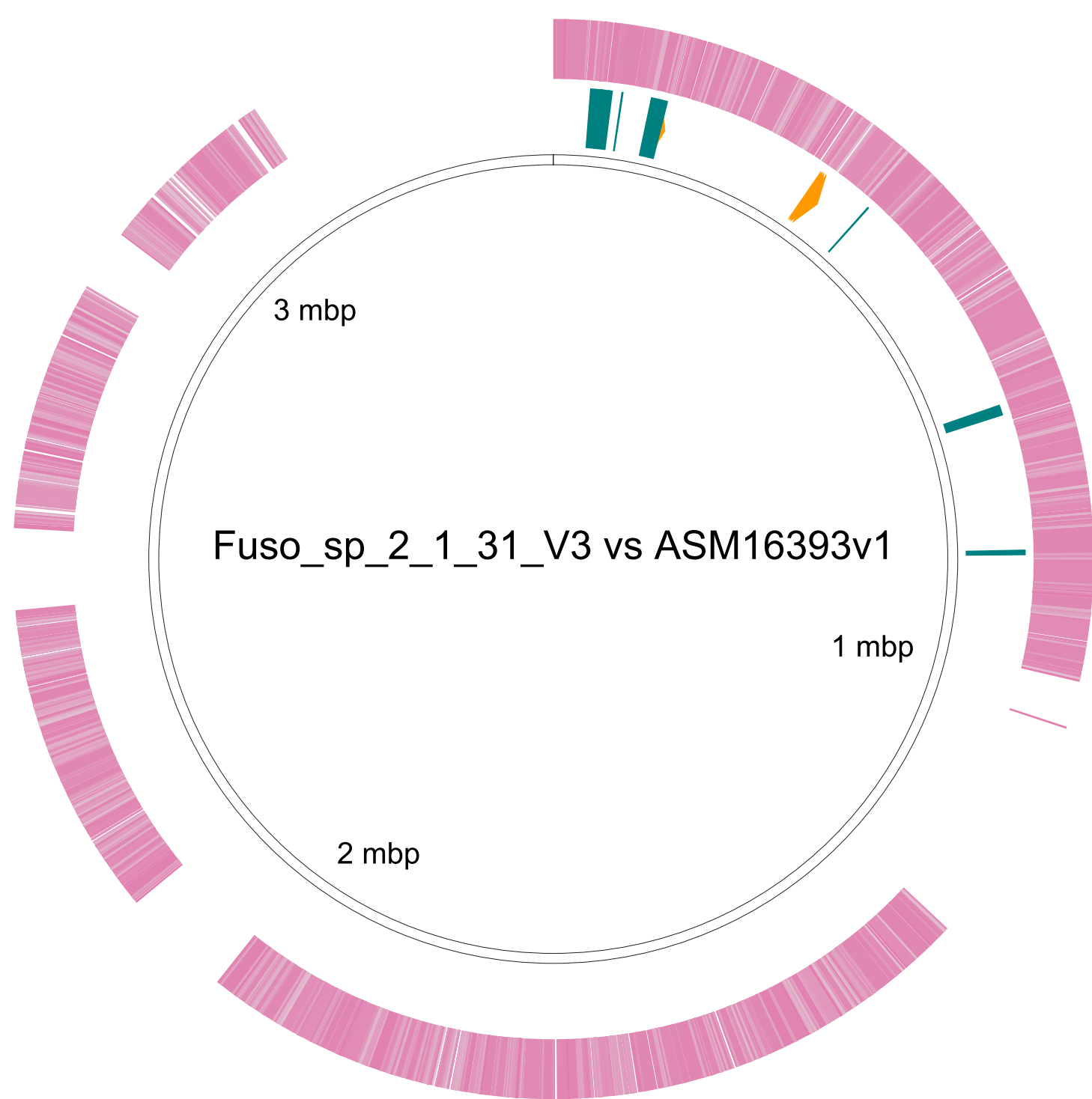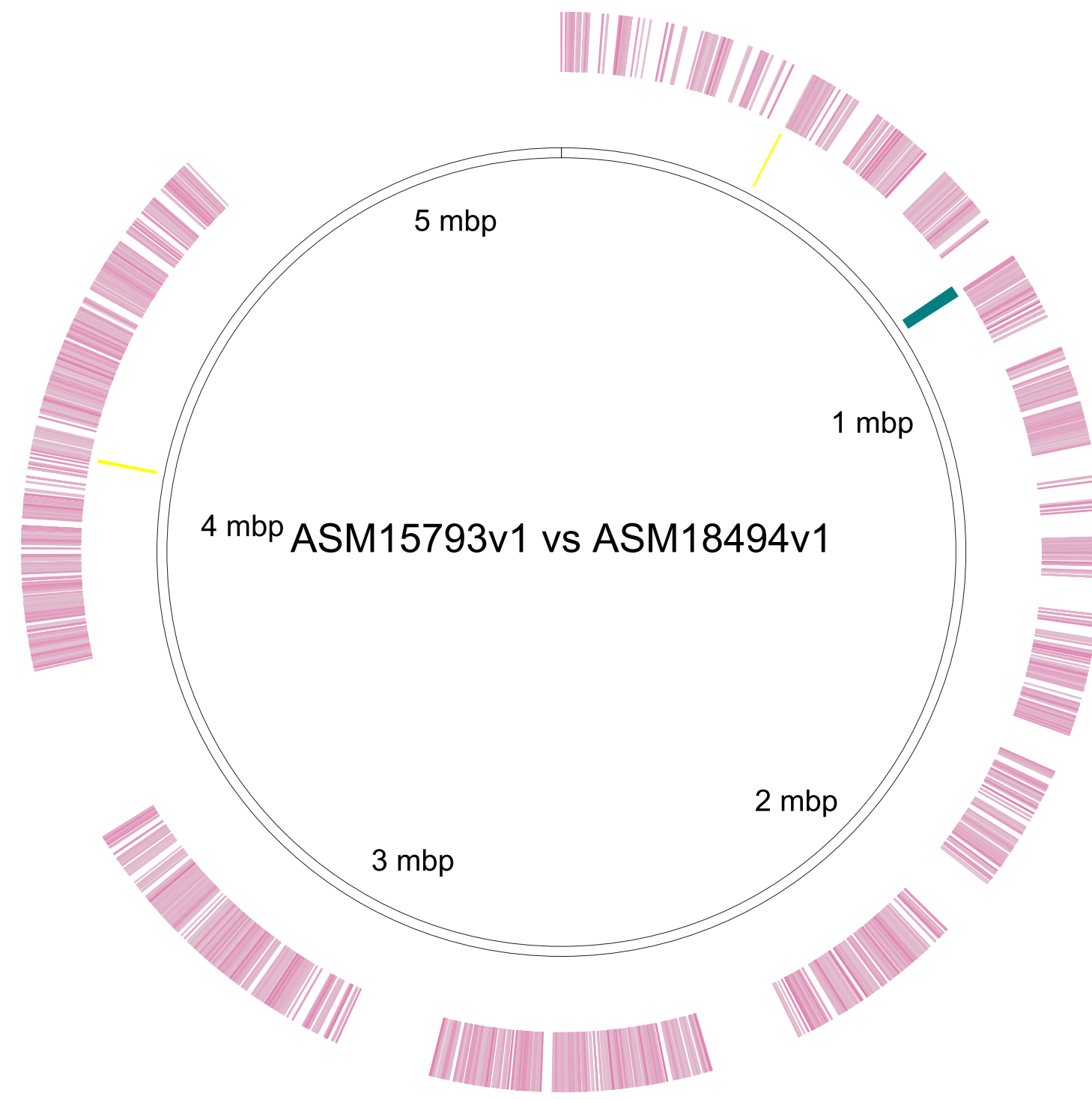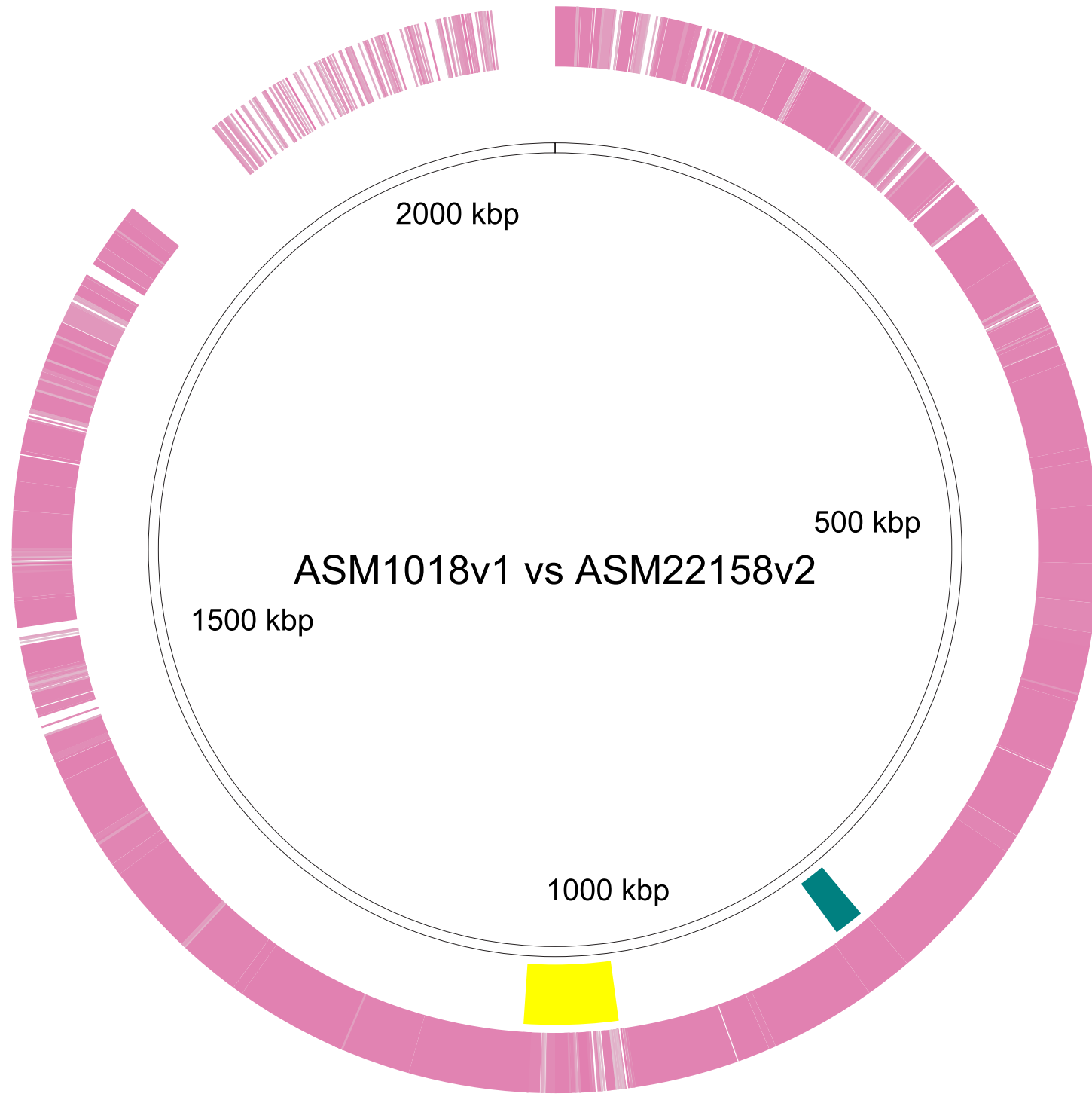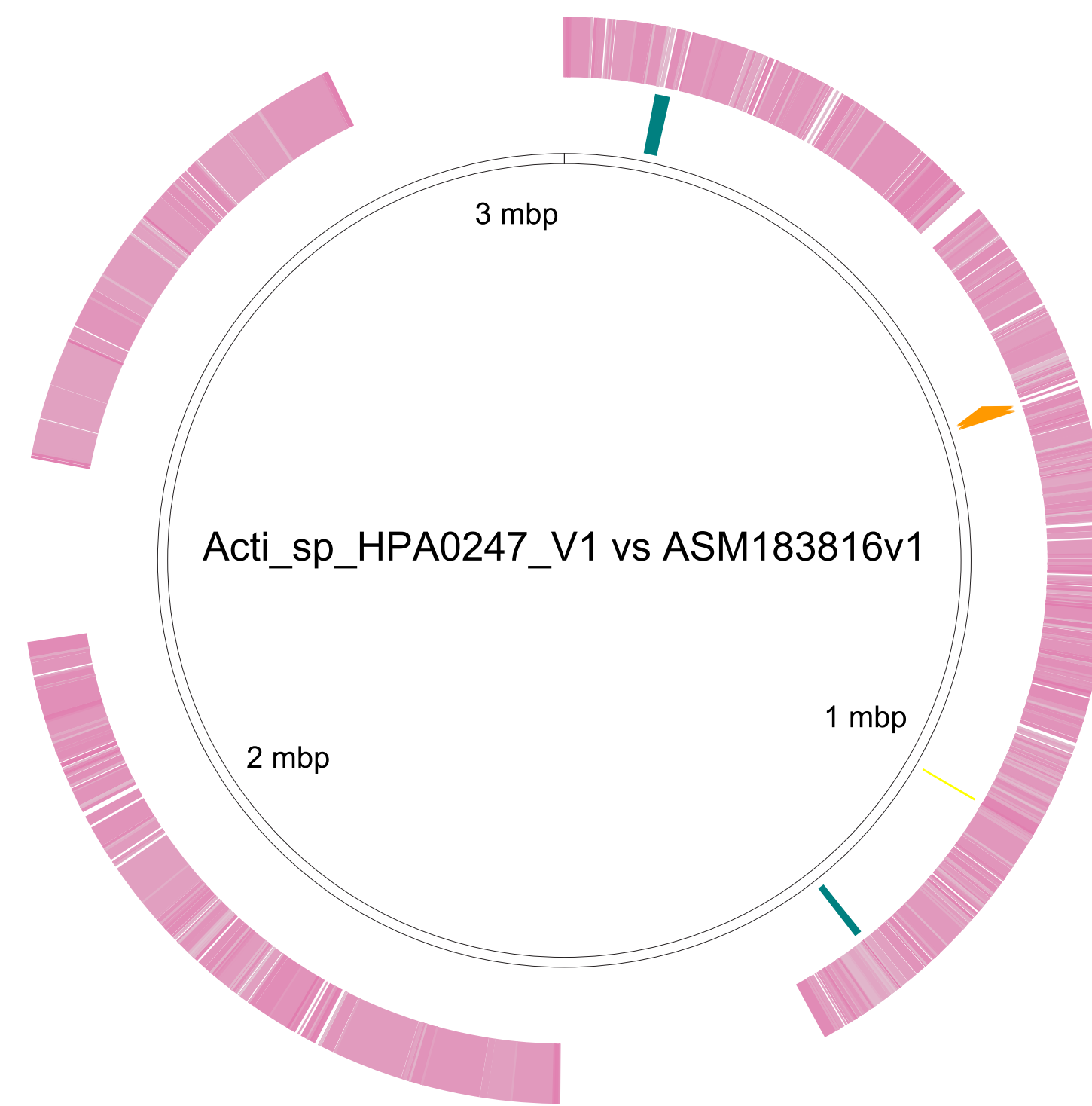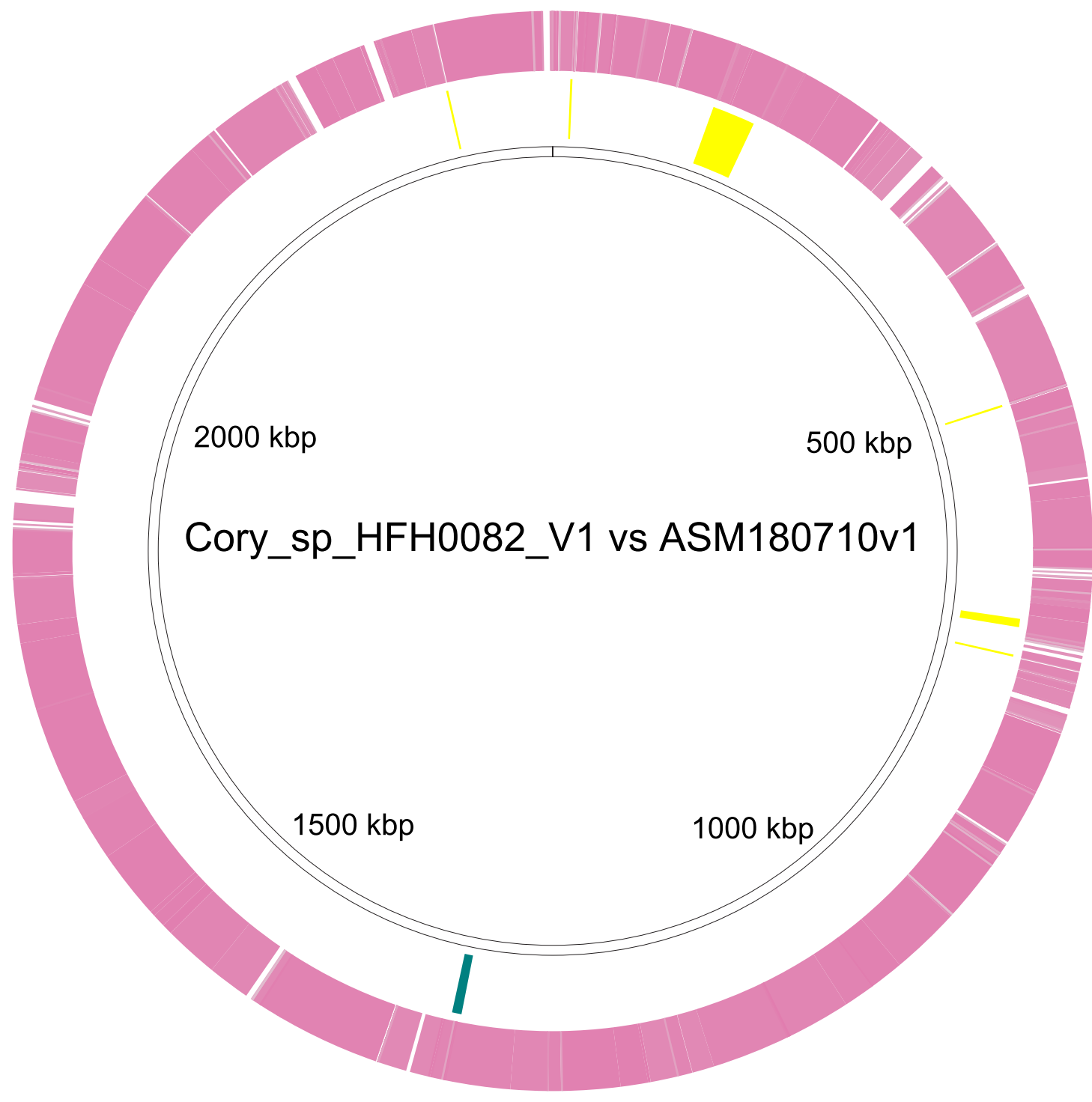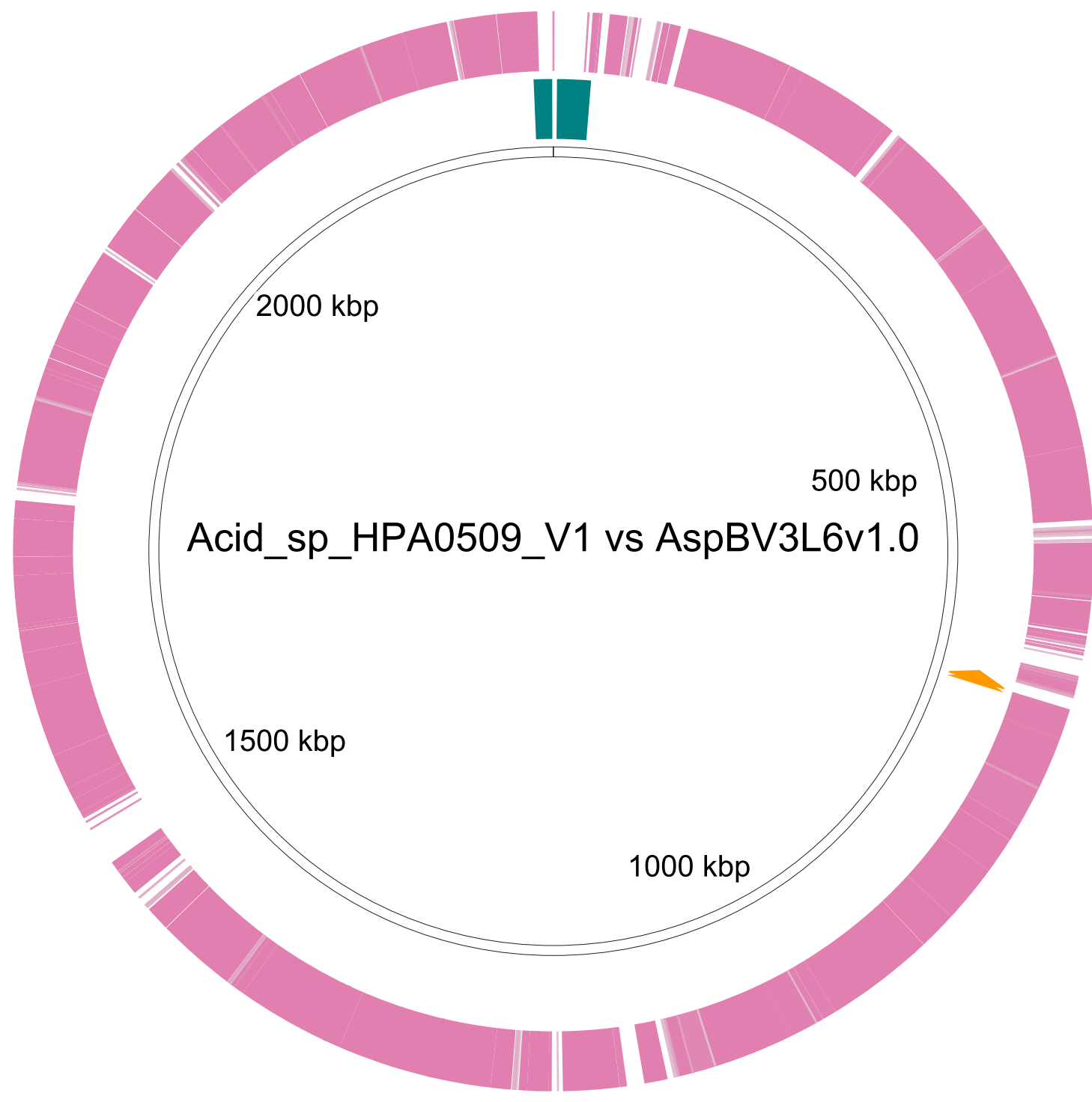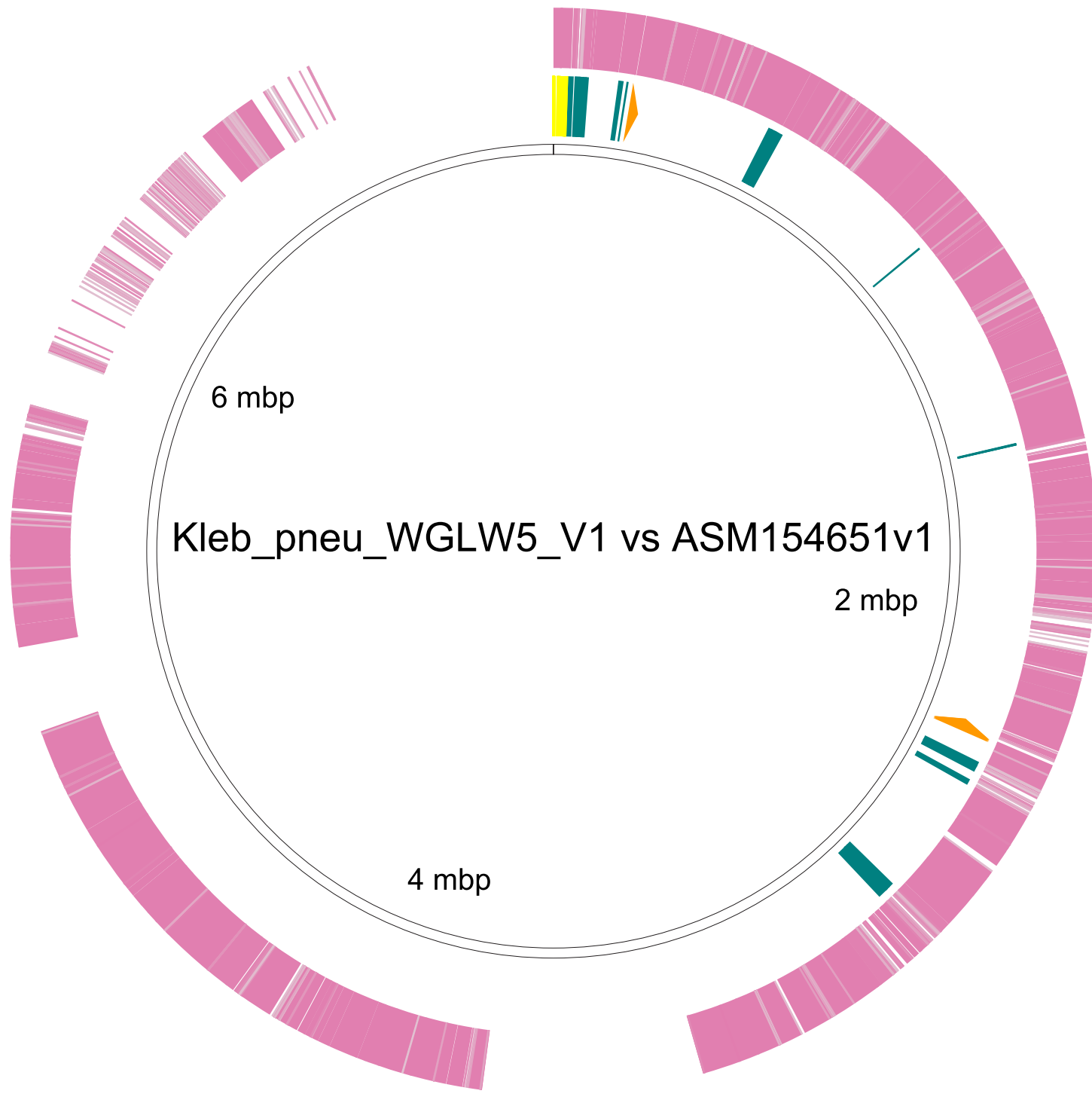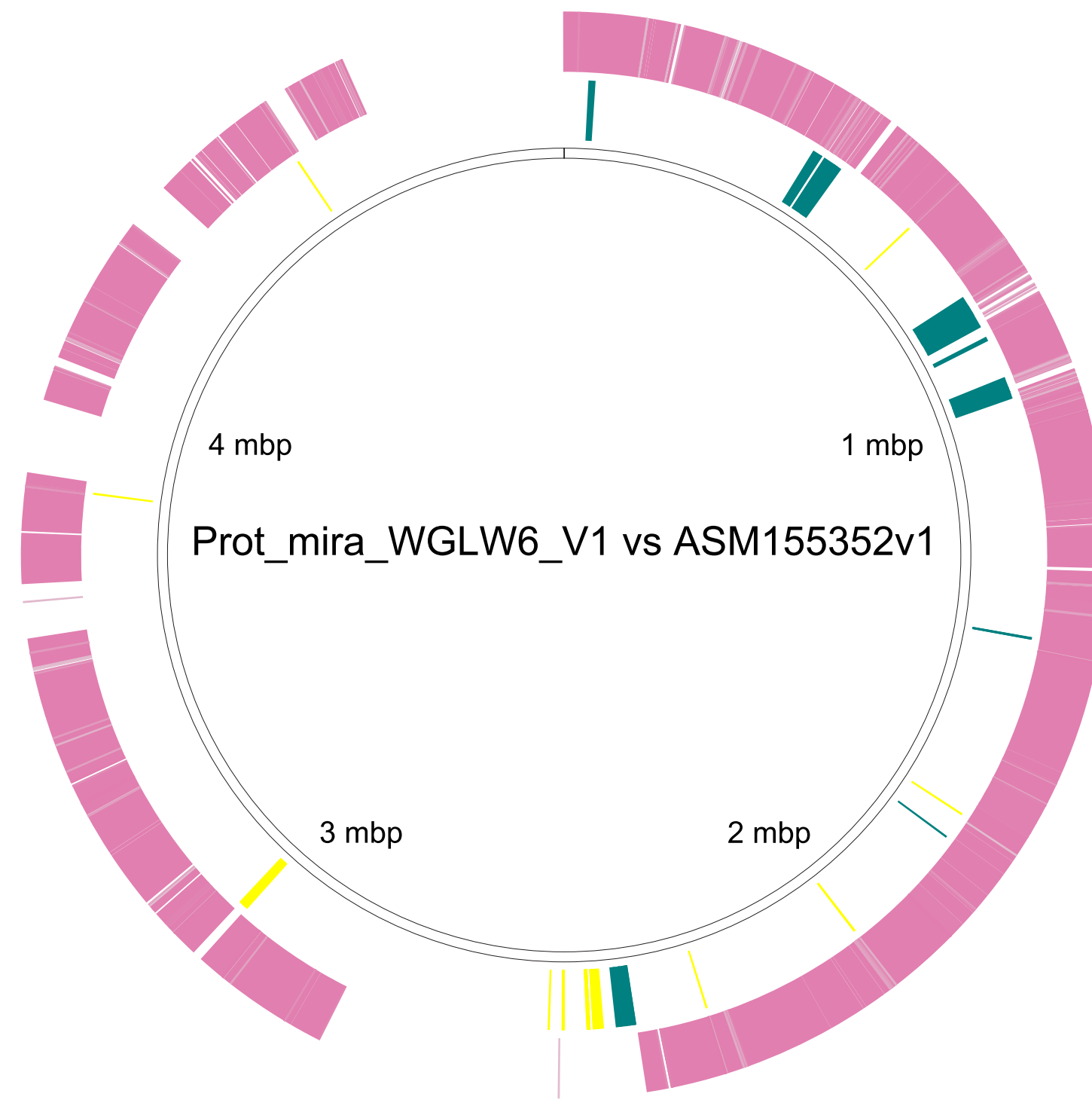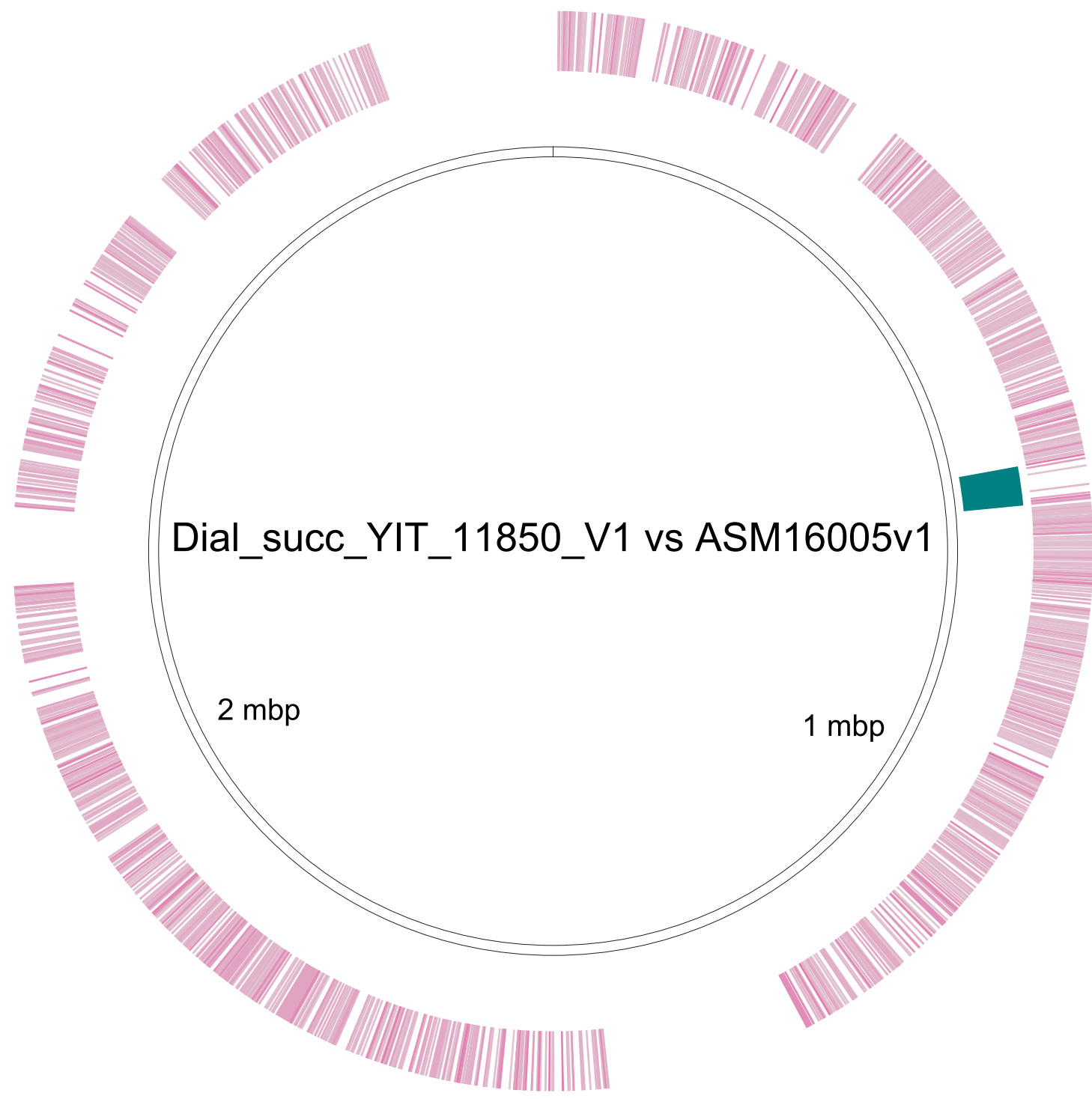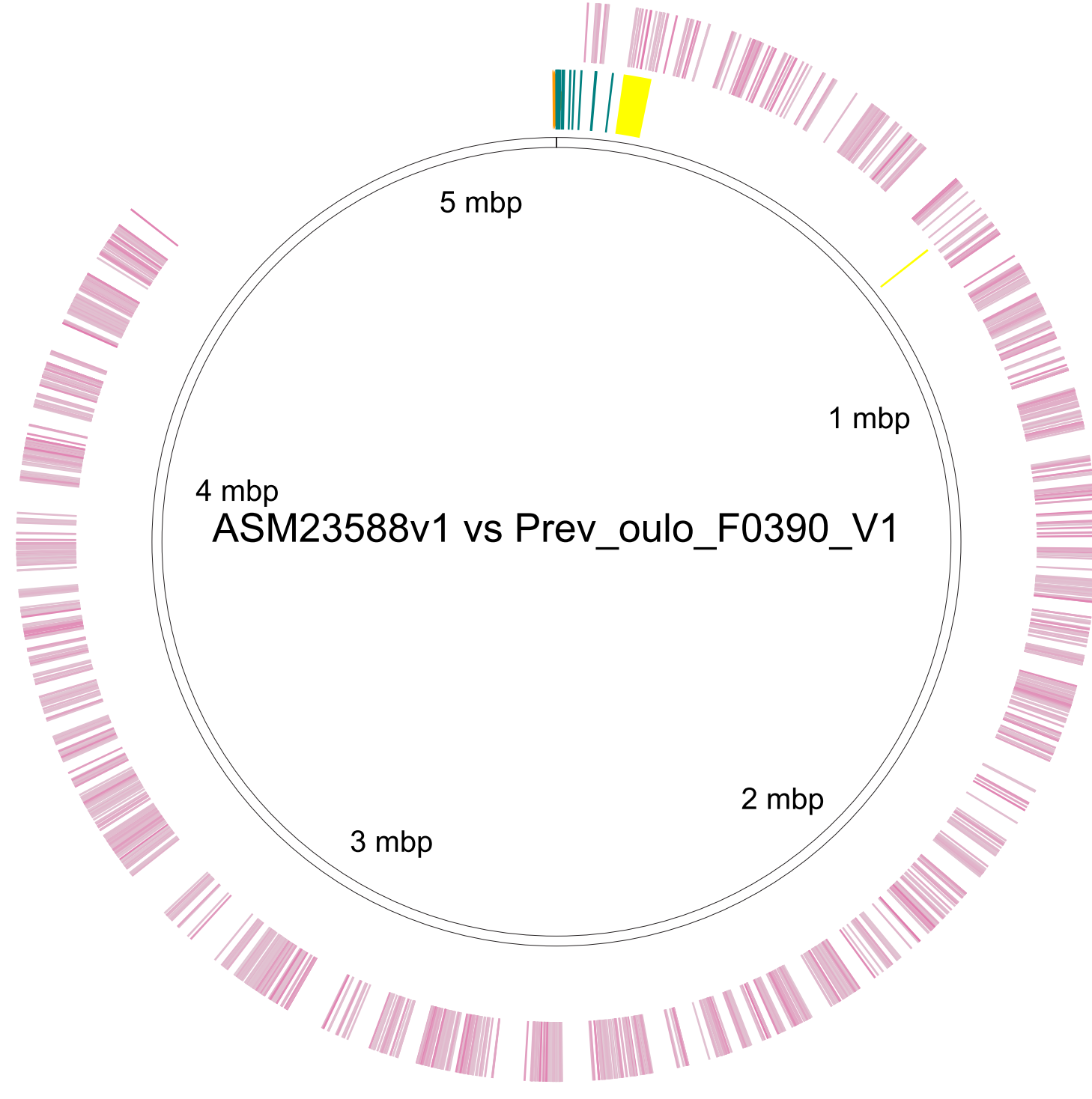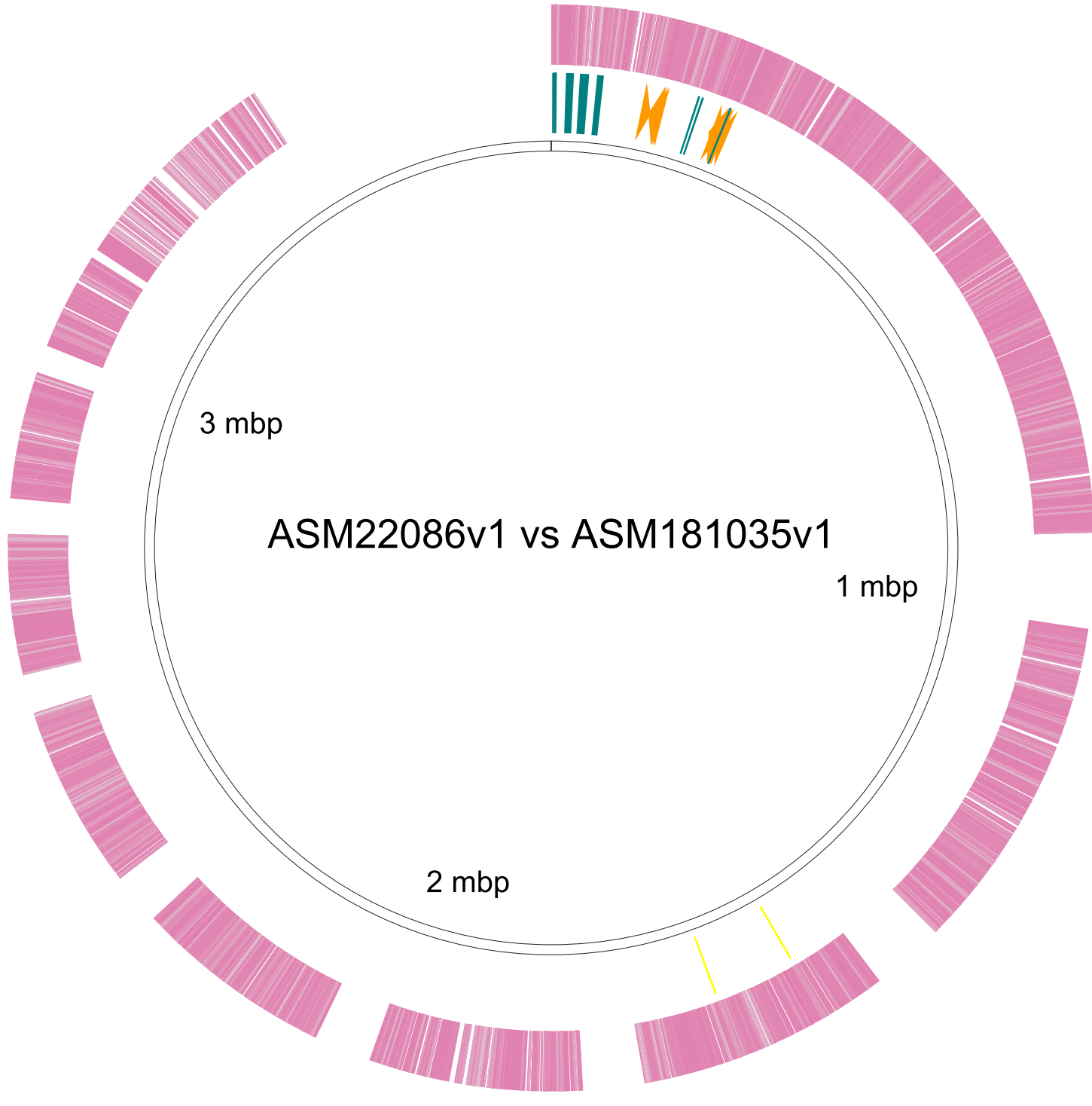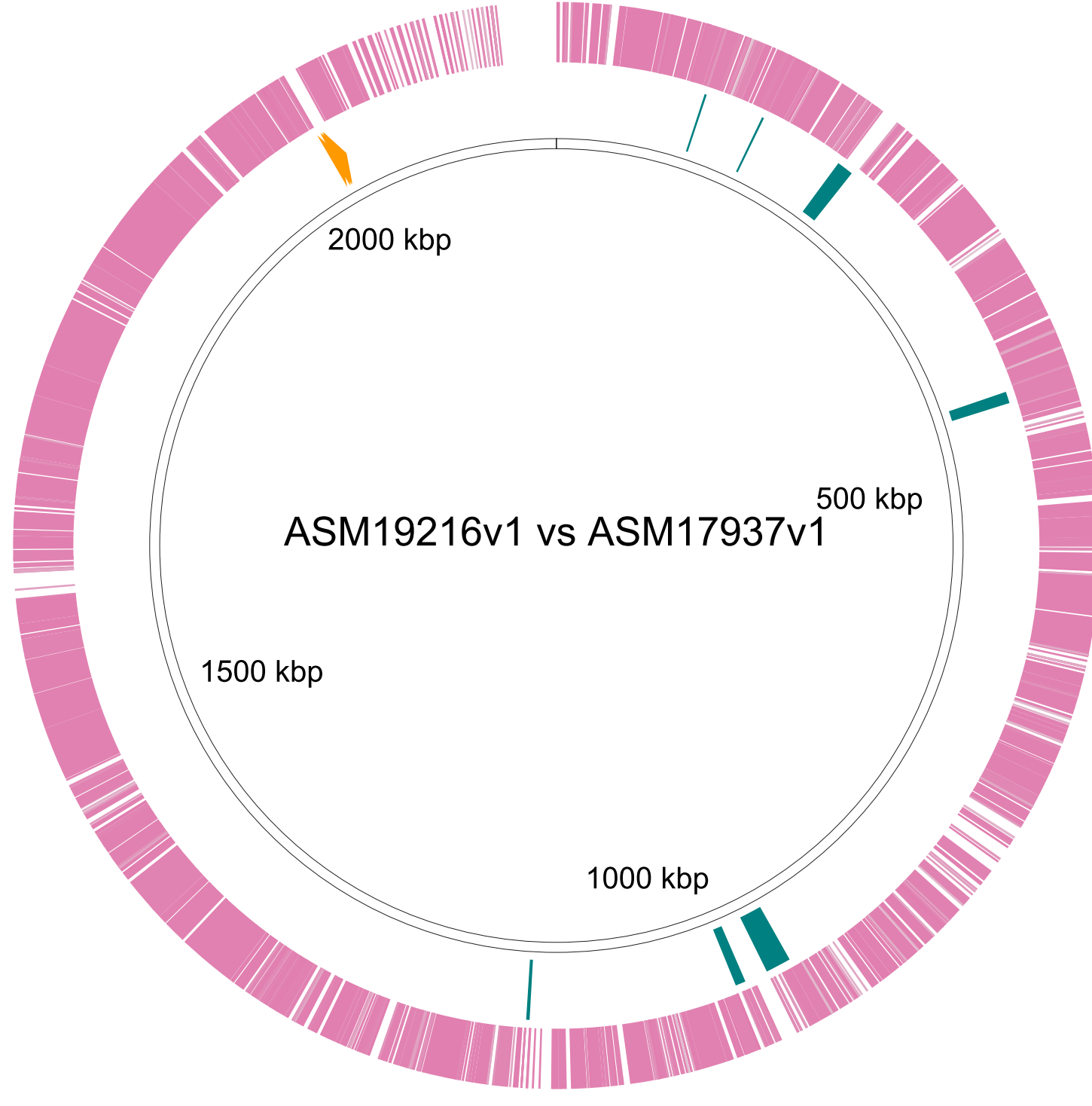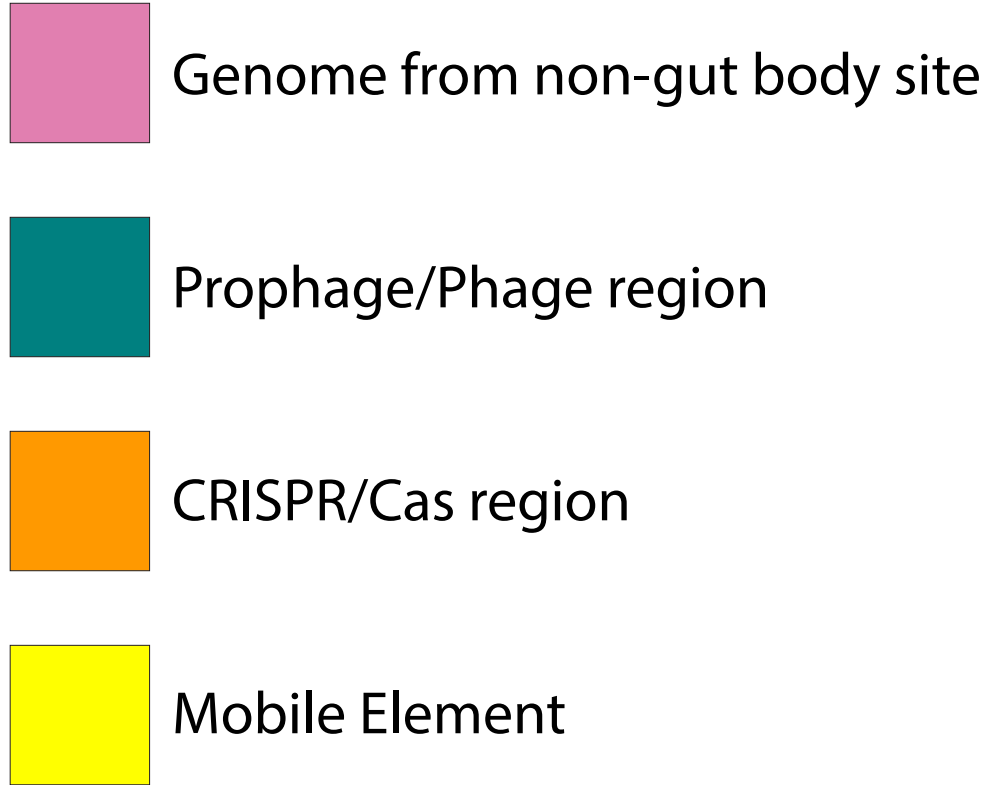

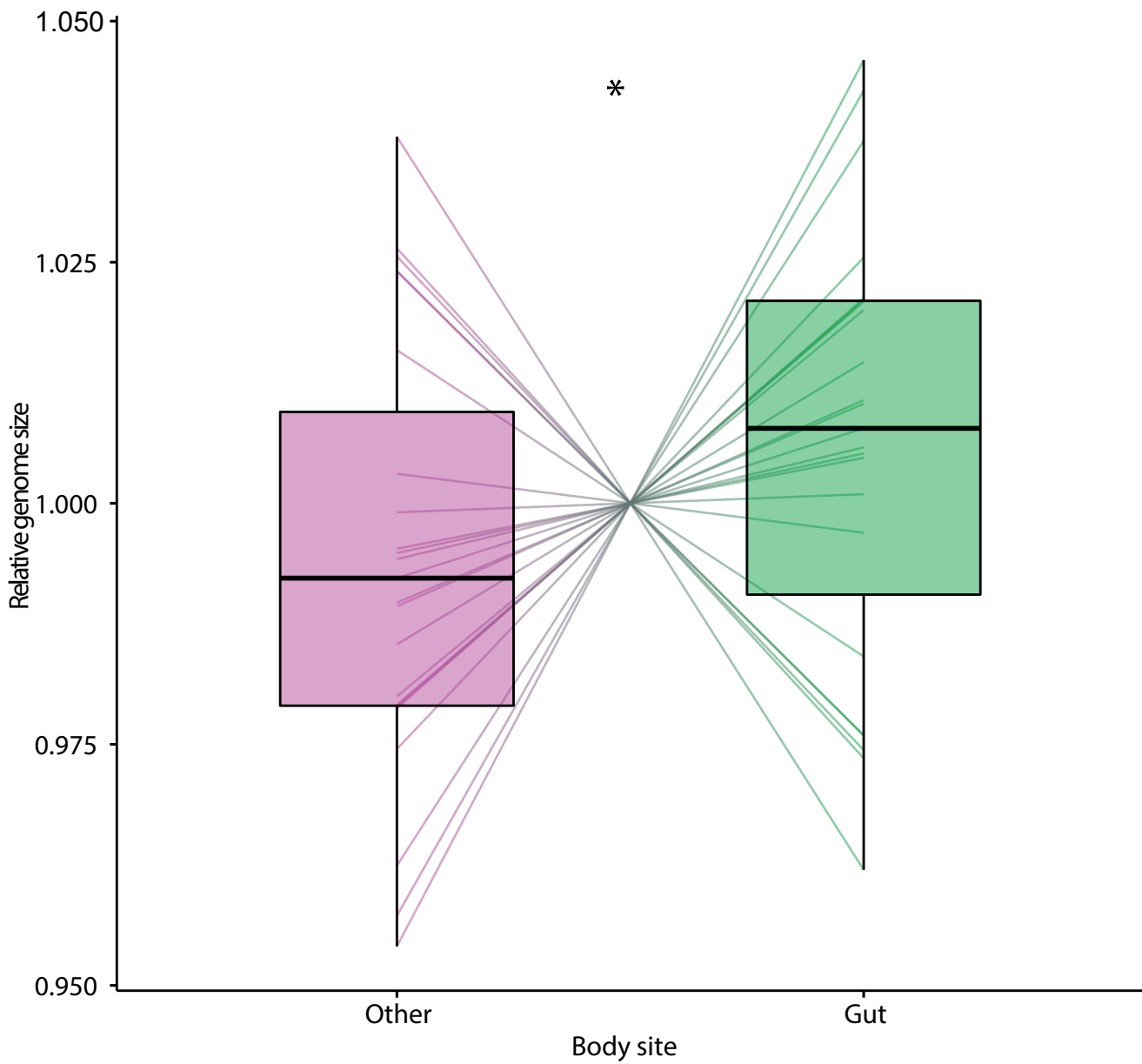

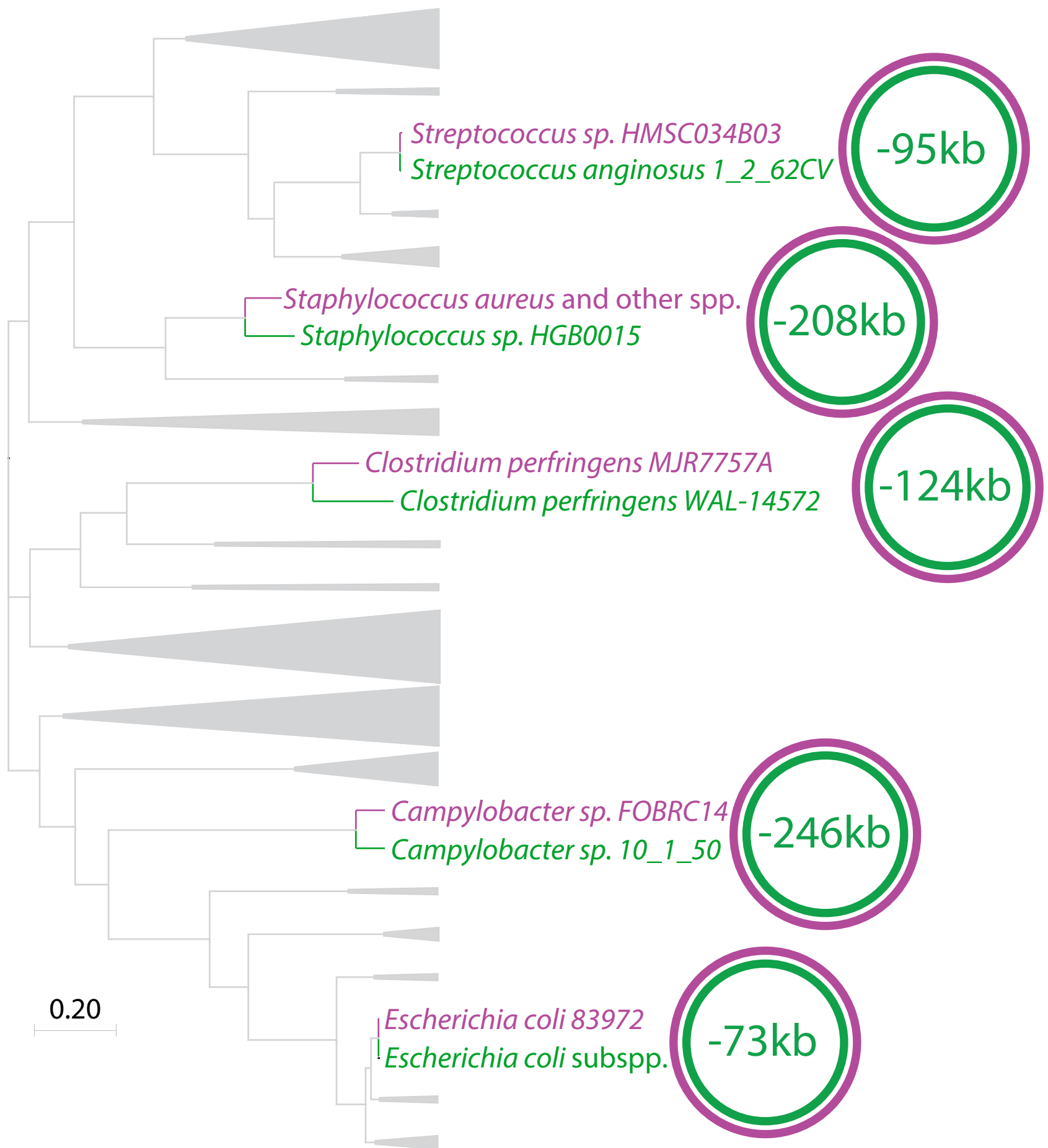

**a**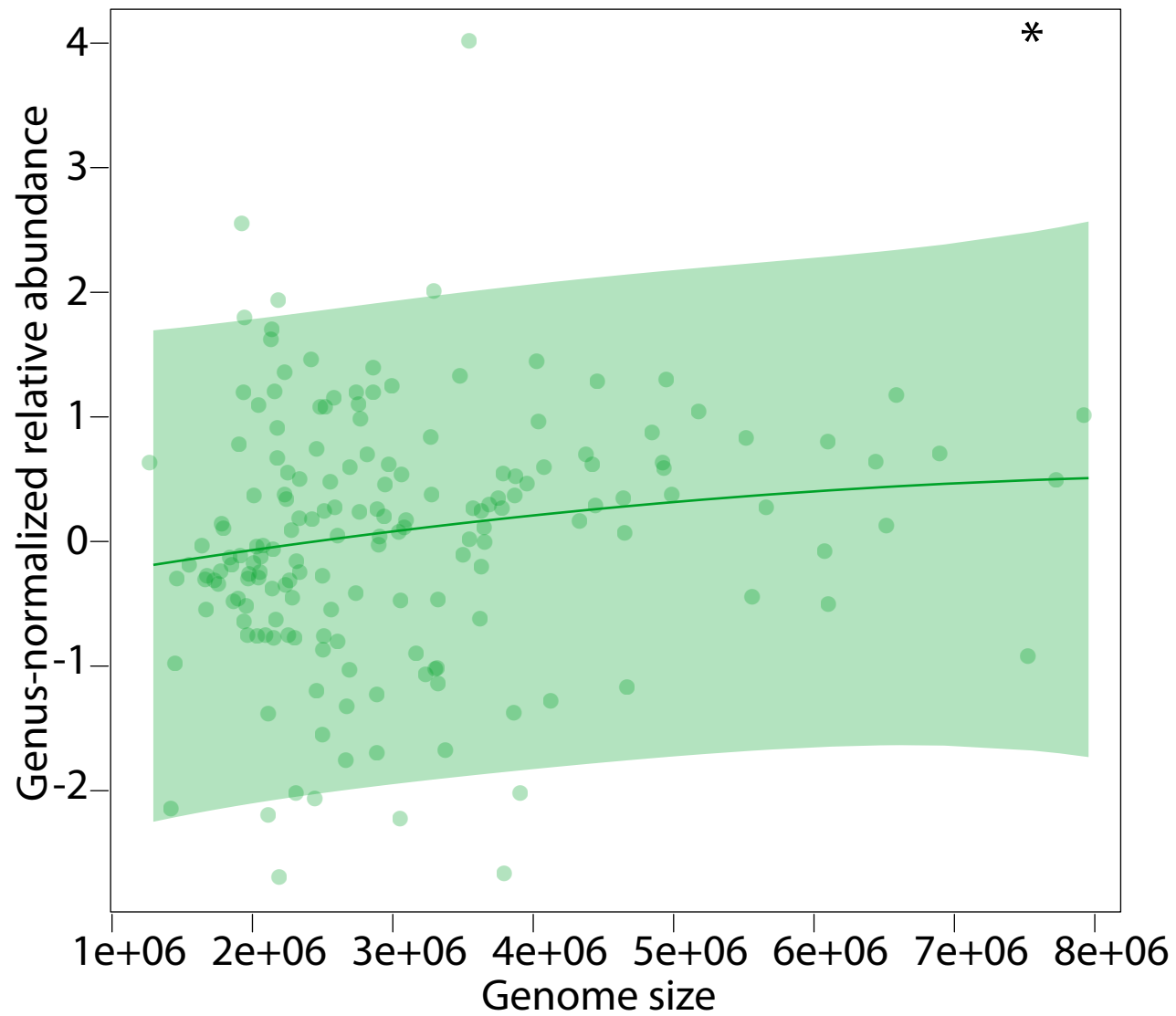**b**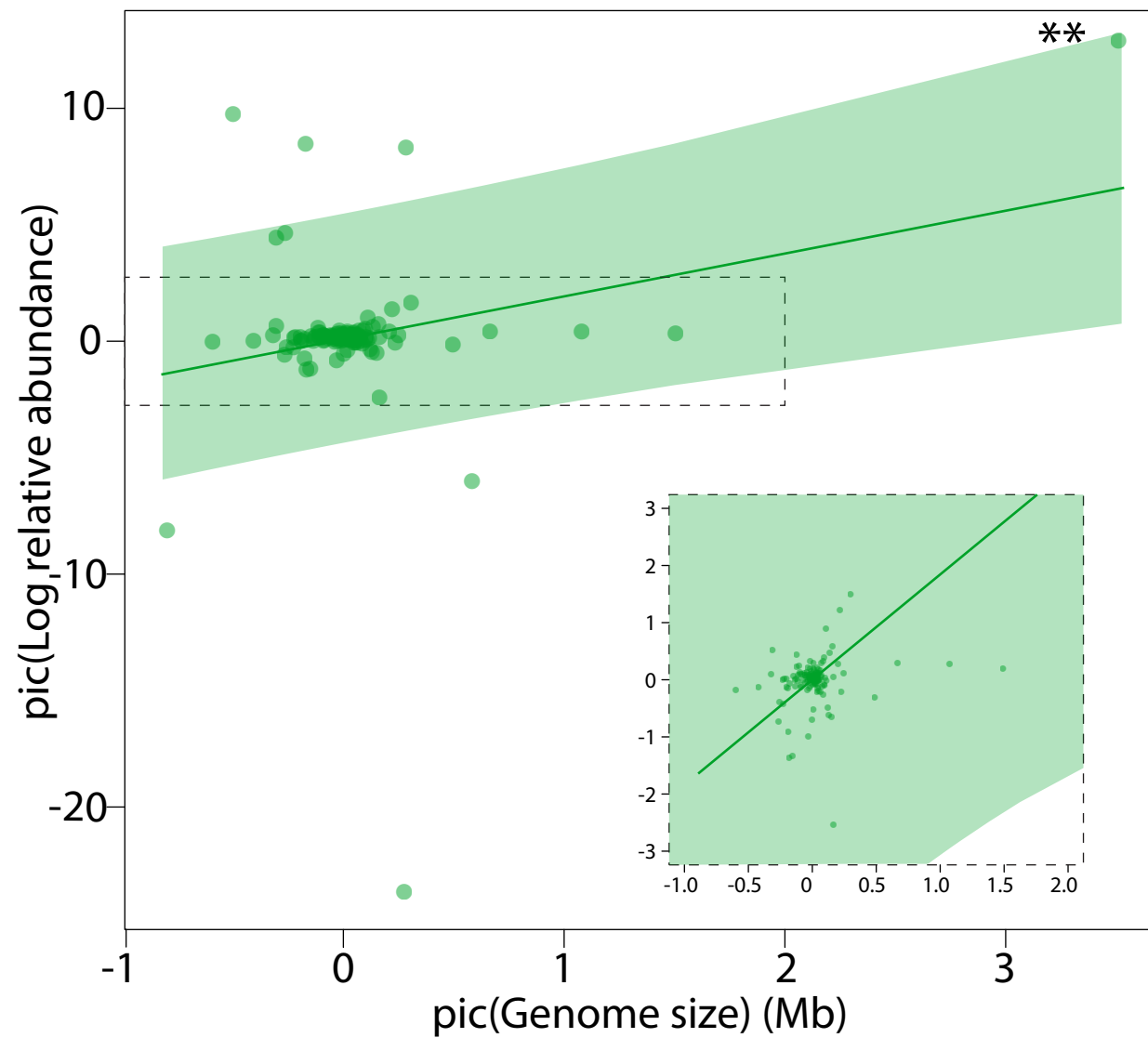

Supplement: evab156_Supplementary_Data [file evab156_supplementary_data.zip › Merged_Supplementary_Figures.pdf]
